# The Hippo effector YAP1/TEAD1 regulates EPHA3 expression to control cell contact and motility

**DOI:** 10.1038/s41598-022-07790-4

**Published:** 2022-03-09

**Authors:** Marwah M. Al-Mathkour, Abdulrahman M. Dwead, Esma Alp, Ava M. Boston, Bekir Cinar

**Affiliations:** 1 Department of Biology and the Center for Cancer Research and Therapeutic Development, Clark Atlanta University, 223 James P. Brawley Dr, SW, Atlanta, GA 30314 USA; 2 Winship Cancer Institute, Emory University, Atlanta, GA USA

**Keywords:** Transcriptional regulatory elements, Cell signalling, Collective cell migration, Cancer

## Abstract

The EPHA3 protein tyrosine kinase, a member of the ephrin receptor family, regulates cell fate, cell motility, and cell–cell interaction. These cellular events are critical for tissue development, immunological responses, and the processes of tumorigenesis. Earlier studies revealed that signaling via the *STK4*-encoded MST1 serine-threonine protein kinase, a core component of the Hippo pathway, attenuated EPHA3 expression. Here, we investigated the mechanism by which MST1 regulates EPHA3. Our findings have revealed that the transcriptional regulators YAP1 and TEAD1 are crucial activators of EPHA3 transcription. Silencing YAP1 and TEAD1 suppressed the EPHA3 protein and mRNA levels. In addition, we identified putative TEAD enhancers in the distal EPHA3 promoter, where YAP1 and TEAD1 bind and promote EPHA3 expression. Furthermore, EPHA3 knockout by CRISPR/Cas9 technology reduced cell–cell interaction and cell motility. These findings demonstrate that EPHA3 is transcriptionally regulated by YAP1/TEAD1 of the Hippo pathway, suggesting that it is sensitive to cell contact-dependent interactions.

## Introduction

The Hippo pathway regulates diverse biological processes, including cell growth, cell contact, cell fate, organ size, development, and carcinogenesis^1,2^. The *STK4*-encoded MST1 and its closest paralog *STK3*-encoded MST2 protein kinase genes are the core components of the Hippo pathway in mammals^2^. Genetic studies in model organisms demonstrated that dysregulation of the Hippo signaling pathway caused developmental defects, immune disorders, and disease^3–6^. For example, silencing hippo or hippo-like MST1 protein resulted in tissue outgrowth in Drosophila^7^ and reduced life span in C. elegans^8^. Likewise, the MST1/2 double knockouts resulted in early embryonic lethality due to excessive cell death, primitive blood vessel formation, and defects in hematopoiesis compared to the single-gene deletion, which had no apparent phenotype^9^. In addition, loss of MST1/2 functions resulted in dilated heart^10–12^, immune cell disorders^13,14^, and malignant cell transformation and cancer, likely due to the overpopulation of tissue-specific stem/progenitor cells^15–17^. Furthermore, the Hippo pathway interacted with multiple signaling networks inside and outside of the cell, suggesting a molecular circuit between the Hippo pathway and other signaling molecules^18–20^.

The transcriptional regulatory protein YAP1 (yes-associated protein 1) and its close paralog WWTR1 are well-characterized nuclear effectors of the Hippo pathway^21,22^. MST1 either alone or through LATS1/2 (large tumor suppressor 1 and 2) phosphorylates and inactivates YAP1 activity^19,23^. The cytoplasmic sequestration and proteasome-mediated degradation caused by phospho-Ser127 modification are essential mechanisms for inhibiting YAP1-mediated gene transcription^15,19^. Genes regulated by YAP1 involve a wide range of cellular biology, including cell–cell interaction, cell fate determination, metabolic processes, and tumorigenesis^24,25^. YAP1 exerts its activity by interacting with transcription factors due to the lack of a DNA binding domain^19,26^. The TEA domain (TEAD) family proteins are well-known transcription factors that mediate the expression of YAP1 target genes^27^.

The erythropoietin-producing human hepatocellular (Eph) receptors are members of the tyrosine kinase receptor (RTK) family that share a highly conserved sequence^28^. The Eph receptors have two subclasses: EphA and EphB^29^. Activation of the Eph receptors by their respective ephrin ligands located at the cell surface could lead to the expression of various genes^30^ that regulate developmental processes^31,32^, viral infections^33^, and cancer^34^. For example, ephrin type-A receptor 3 (EPHA3), a member of the EphA receptor subclass, controls cell fate, cell shape, cell communication, axon guidance, and the embryonic development of vital organs like the brain, heart, lungs, and kidney^35–38^. For example, EPHA3 signaling mediates elongation and navigation of axons and trajectories and the assembly of spinal motor neuron axons^39,40^. In addition, a recent study has suggested that EPHA3/ephrin-A5 signaling limits axon development and governs axon guidance in developing neurons^41^. Also, EPHA3 could modulate cell migration and neurite outgrowth^42^, likely by controlling actin dynamics^43^ through CrkII and RhoA signaling^44^. Moreover, increasing evidence suggests that dysregulated EPHA3 signaling is implicated in multiple malignancies with poorer prognosis^45–48^. Despite these observations, however, little is known about the mechanism contributing to the transcriptional regulation of EPHA3.

An earlier study from our laboratory suggested that MST1/STK4 signaling suppressed EPHA3 expression^49^; however, the underlying mechanism is unknown. The present study showed that the YAP1 and TEAD1 proteins are potent activators of EPHA3 expression. Our data have demonstrated that YAP1 and TEAD1 activate the EPHA3 promoter by binding to the putative TEAD responsive elements (TREs) located in the EPHA3 distal promoter. Furthermore, we have demonstrated that loss of EPHA3 signaling significantly reduced cell–cell interaction and cell motility. Thus, to our knowledge, this is the first study showing that the YAP1 and TEAD1 proteins transcriptionally regulate EPHA3 expression and its cellular biology downstream of the Hippo pathway.

## Results

### MST1 signaling attenuates EPHA3 expression

**Figure 1:**
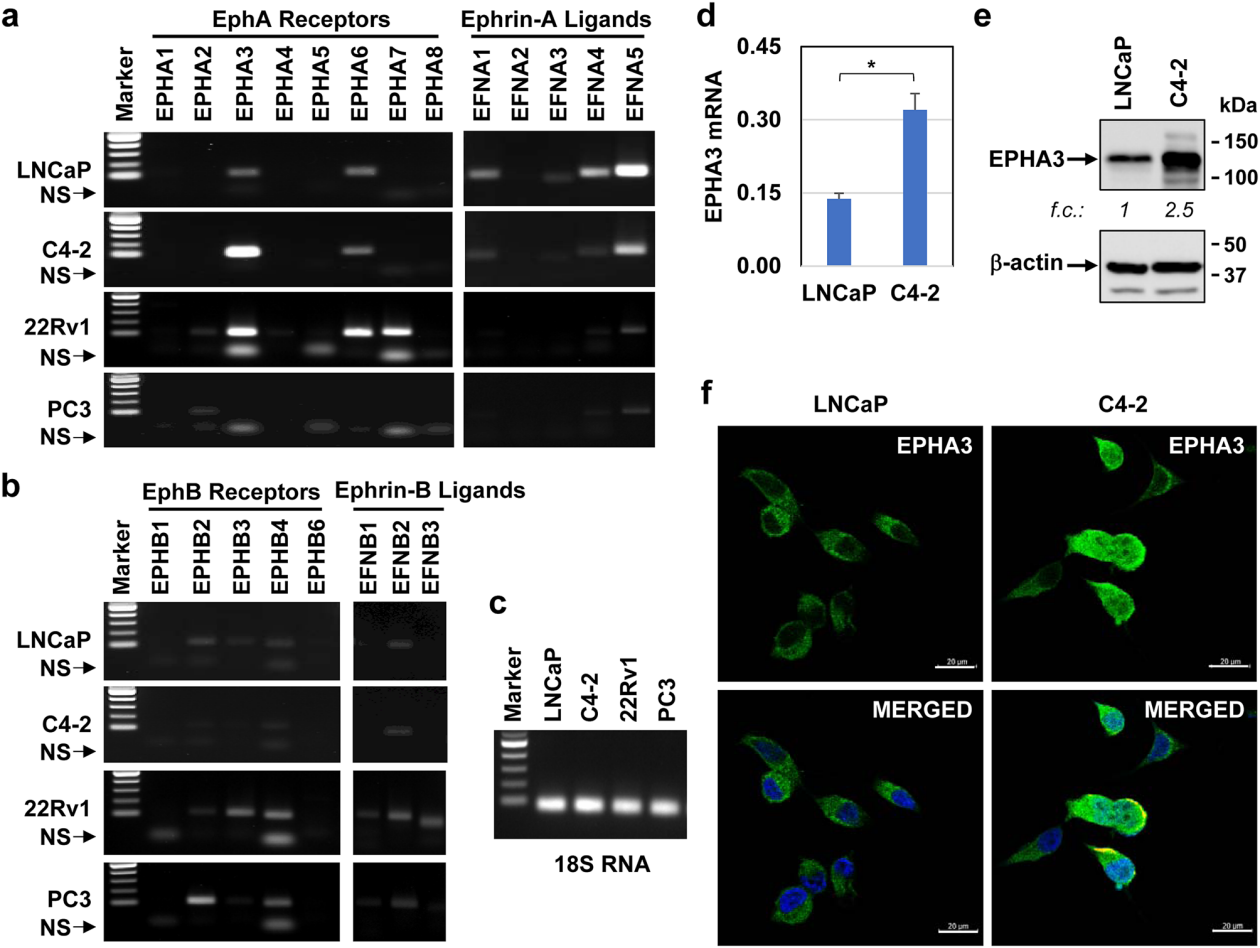
Expression of Ephrin A and B receptors and their ligands in the cell lines. (**a, b**) Transcripts of EphA and EphB receptors and their ligands in LNCaP, C4-2, 22Rv1, and PC3 prostate cancer cell lines. NS: nonspecific bands (**c**) 18S RNA was included as an internal control in PCR reactions. The PCR product was resolved on a 1.5% agarose gel and imaged using the DNA gel documentation system. (**d**) Quantitative PCR analysis of EPHA3 transcripts, *P < 0*.*01. (**e**) Western blot (WB) analysis of EPHA3 protein in whole-cell lysates. The β-actin protein blot was included as an internal control in the WB. (**f**) Immunofluorescence imaging of EPHA3 protein in fixed cells. Cells were grown under steady-state conditions. Micrographs represent at least three independent experiments—size bars: 20 µm.

To determine the mechanism by which STK4/MST1 signaling controls EPHA3 expression, we first assessed the abundance of the Ephrin A (EphA) and Ephrin B (EphB) receptor subtypes and their respective ligands in the well-characterized human prostate cancer cell lines LNCaP, C4-2, 22Rv1, and PC3. The results showed that LNCaP, C4-2, 22Rv1, and PC3 cell differentially expressed EphA and EphB receptors and their ligands, as demonstrated by RT-PCR (Fig. 1a–c; Figure S1 and S2). Notably, the levels of *EPHA3* and its ligand, ephrin-A5 (encoded by the *EFNA5* gene), were abundant in LNCaP and C4-2 cells compared to other EphA receptors. In addition, *EPHA3* transcript levels were markedly higher in C4-2 than in LNCaP cells (Fig. 1a). In contrast, the levels of *EPHA1*, *EPHA2*, *EPHA4*, *EPHA5*, *EPHA7*, and *EPHA8* transcripts were very low or undetectable in LNCaP and C4-2 (Fig. 1a). Similarly, *EFNA5* transcript levels were higher in LNCaP and C4-2 cells than other ephrin-A ligands (*EFNA1-4*) (Fig. 1a). On the other hand, 22Rv1 expressed the high *EPHA3*, *EPHA6*, and *EPHA7* mRNA levels relative to the *EPHA1*, *EPHA2*, *EPHA4*, and *EPHA5* (Fig. 1a). In contrast, *EPHA1*, *EPHA4*, *EPHA5*, *EPHA6*, *EPHA7*, and *EPHA8* mRNA levels, except for *EPHA2*, were essentially undetectable in the PC3 line (Fig. 1a). In addition, 22Rv1 and PC3 cells expressed very low *EFNA4/5* transcript, whereas the expression of *EFNA1-3* was undetectable (Fig. 1a). Nevertheless, the expression of EphB receptors and ephrin-B ligands, compared to EphA receptors and ephrin-A ligands, were low in LNCaP, C4-2, 22Rv1, and PC3 cells (Fig. 1b).

**Figure 2:**
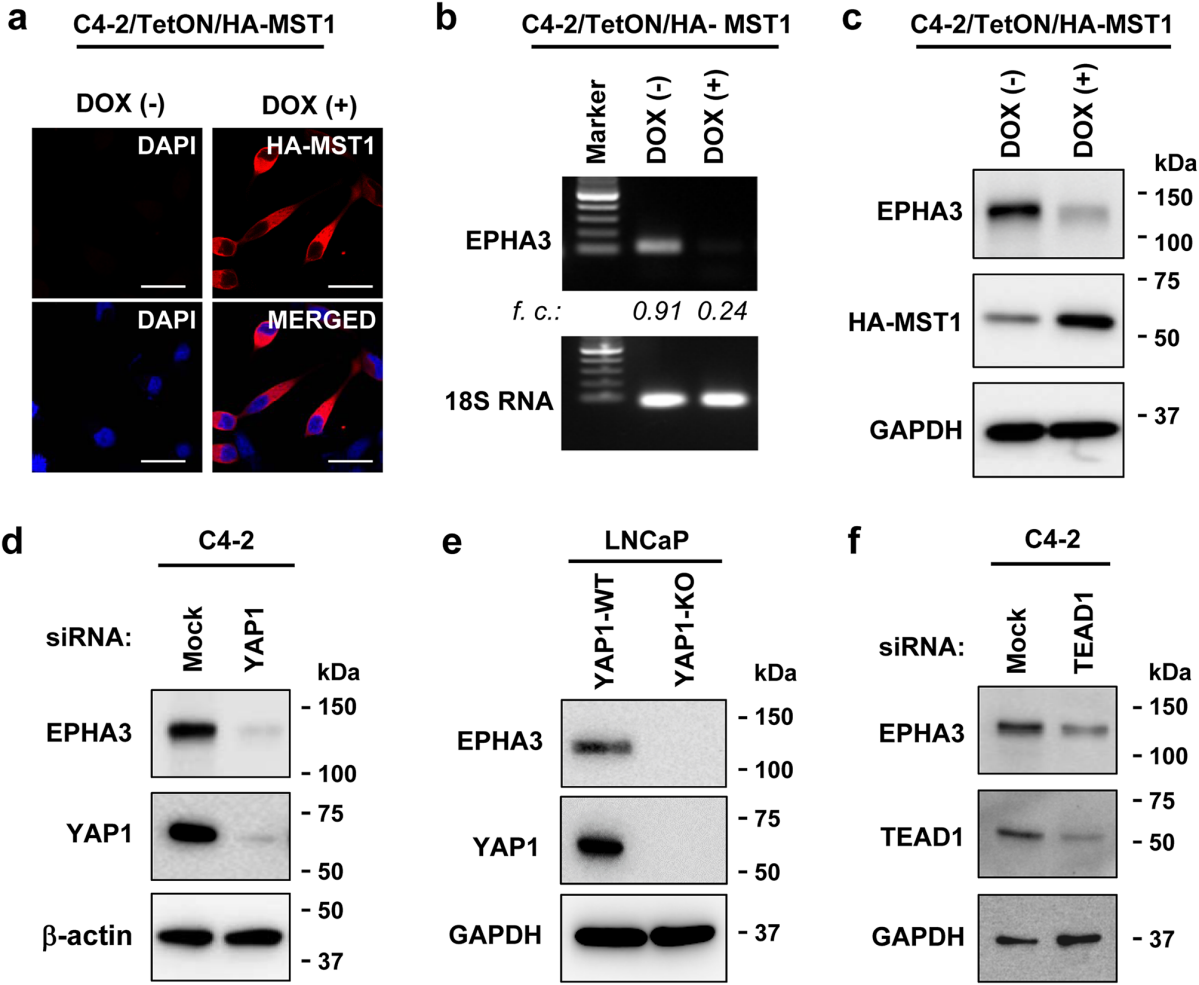
Regulation of EPHA3 expression by MST1-YAP1-TEAD1 signaling. (**a**) Induction of ectopic HA-tagged MST1 protein, as assessed by immunofluorescence imaging. (**b**) RT-PCR analysis of EPHA3 transcripts. 18S RNA was used as an internal control in the PCR reaction. (**c**) WB analysis of EPHA3 and HA-tagged MST1/STK4 protein. Total RNA and proteins were isolated from C4-2/TetON/HA-MST1 cells after treatment with (+) or without (−) doxycycline (Dox, 2 µg/ml) in TetON-approved serum-fed condition. (**d-f**) EPHA3 protein levels in LNCaP or C4-2 cell lines with or without YAP1 or TEAD1 silencing. Membrane probed with the protein-specific antibody. The β-actin or GAPDH protein blot was used as an internal control in WB. Size bars: 20 µm. Data are the representation of three independent experiments.

Moreover, our quantitative PCR showed that *EPHA3* mRNA levels were twofold higher in C4-2 than in LNCaP cells (Fig. 1d), consistent with the RT-PCR results above. Similarly, compared with the LNCaP, increases in *EPHA3* mRNA levels in C4-2 cells correlated with augmented EPHA3 protein expression, as assessed by western blotting (Fig. 1e; Fig. S3) and immunofluorescence imaging (Fig. 1f). Furthermore, we showed that relative to the control, controlled induction of MST1/STK4 expression in the engineered C4-2 cell line (Fig. 2a) reduced the levels of *EPHA3* mRNA by about 75% (Fig. 2b; Fig. S4b), which coincided with the reduction in EPHA3 protein levels (Fig. 2c; Figure S4b).

### YAP1 and TEAD1 regulate EPHA3 expression

MST1 signaling suppresses YAP1 activity by promoting inhibitory phospho-Ser127 modification and cytoplasmic sequestration^49^. We evaluated EPHA3 protein levels in C4-2 cells with and without YAP1 knockdown conditions. Silencing YAP1 by siRNA downregulated EPHA3 protein expression relative to mock control siRNA (Fig. 2d; Fig. S5). Similarly, YAP1 knockout attenuated EPHA3 protein compared with the YAP1-WT (Fig. 2e; Fig. S6). The TEAD transcription factors are critical mediators of the YAP1 transcriptional activity, and conversely, YAP1 constitutes an essential component of the TEAD-dependent gene expression^50^. In this regard, silencing TEAD1 by siRNA reduced EPHA3 expression by half compared with the mock siRNA control (Fig. 2f; Fig. S7). Interestingly, however, silencing TEAD2 and TEAD3 did not affect EPHA3 expression (not shown). These findings suggest that the YAP1-TEAD1 axis likely transcriptionally regulates EPHA3.

### YAP1 and TEAD1 bind EPHA3 promoter

**Figure 3:**
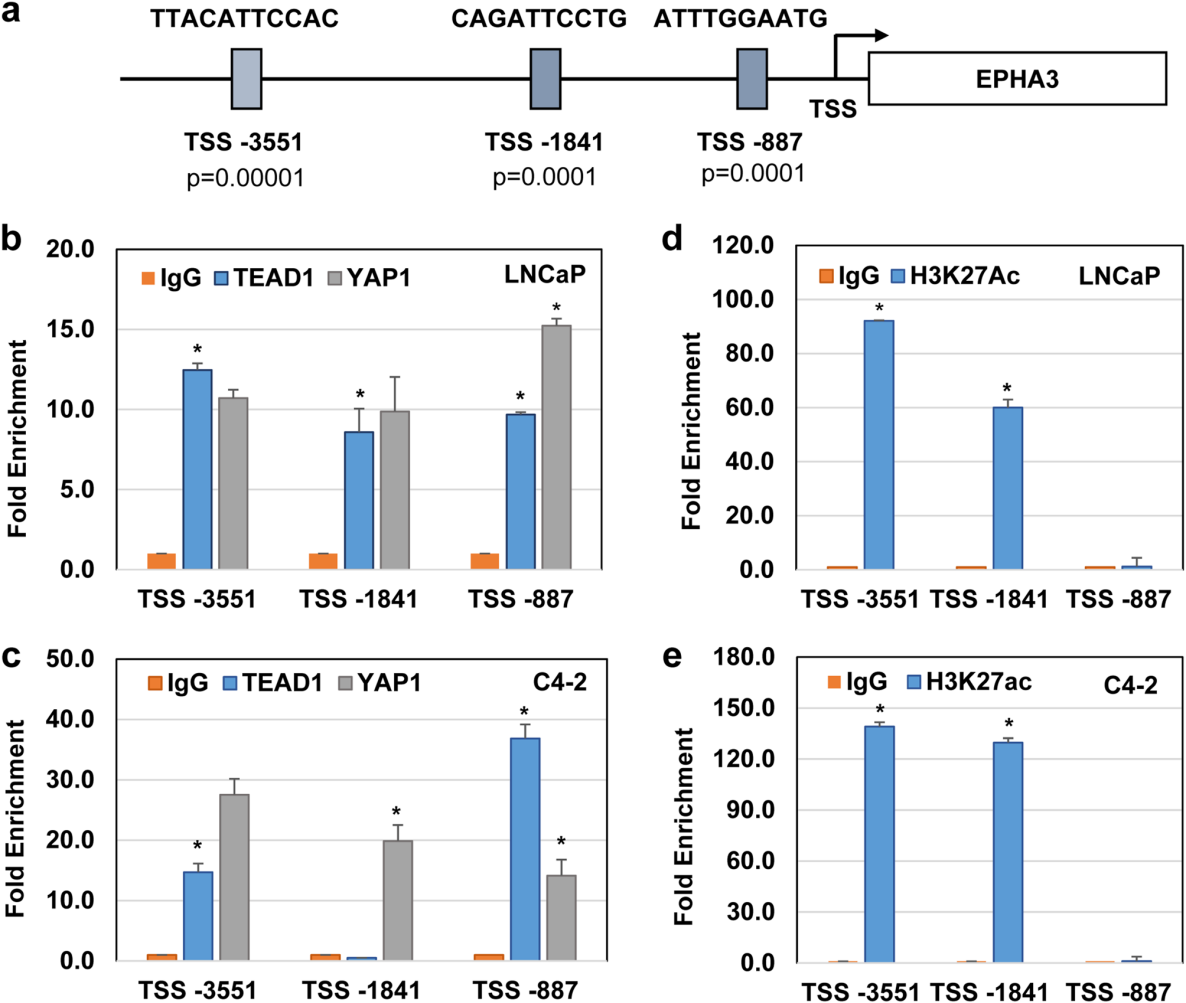
Presence of TEAD1 binding sides located in the EPHA3 promoter. (**a**) Schematic representation and location of the TEAD responsive elements (TREs) within the -5 kb DNA region of the EPHA3 promoter relative to the transcriptional start site (TSS). The JASPAR and EPD bioinformatics tools were employed to identify TREs. (**b-e**) ChIP-qPCR analysis of protein-crosslinked genomic DNA fragments bound by IgG or ChIP-grade TEAD1, YAP1, or H3K27Ac antibodies; *P < 0.001. Protein-crosslinked and sonicated DNA fragments were isolated from LNCaP or C4-2 cells that were grown in serum-fed conditions. The bound and eluted DNA fragments were analyzed by qPCR using a primer set amplifying the indicated TRE regions, and the results were normalized to the IgG control. Data are the representation of three independent experiments.

To understand the mechanism by which YAP1 and TEAD1 regulate EPHA3 expression, we computationally scanned the -5 kb of EPHA3 promoter relative to transcriptional starts site (TSS) for potential TEAD responsive elements (TREs) using EPD^51^, JASPAR^52^, or ConSite^53^ transcription factor binding site databases. The scanning let us identify three highly statistically significant putative TREs, named TSS–3351 (*P* < 0.00001), TSS–1841 (*P* < 0.0001), and TSS–887 (*P* < 0.0001) (Fig. 3a). To determine whether YAP1 and TEAD1 occupy the computationally identified TREs, we conducted a chromatin immunoprecipitation (ChIP) assay. When combined with qPCR, ChIP assay is a powerful method to evaluate protein-DNA interaction^54^. qPCR demonstrated that compared to the IgG control, YAP1 and TEAD1 occupied TREs at the TSS–3551, TSS–1841, and TSS–887 DNA regions in LNCaP (Fig. 3b). Surprisingly, although YAP1 interacted with all three TREs in C4-2, TEAD1 interacted with only TSS–3551 and TSS–887, but not TSS–1841, in C4-2 cells (Fig. 3c).

Acetylation of Histone 3 at lysine 27 (H3K27ac) is a well-characterized enhancer marker^55^. Therefore, to determine which TREs serve as an enhancer for TEAD1, we repeated the ChIP experiment with the H3K27ac antibody and analyzed the H3K27ac-bound DNA fragment by qPCR. The results showed that H3K27ac abundantly occupied the TSS–3351 and TSS–1841 regions but without occupying the TSS–887 in LNCaP and C4-2 cells (Fig. 3d,e, respectively). This observation is specific because the IgG control did not show any enrichment in all three sites, indicating that TSS–3551 acts as a putative enhancer for TEAD1.

### YAP1 and TEAD1 activate EPHA3 promoter

**Figure 4:**
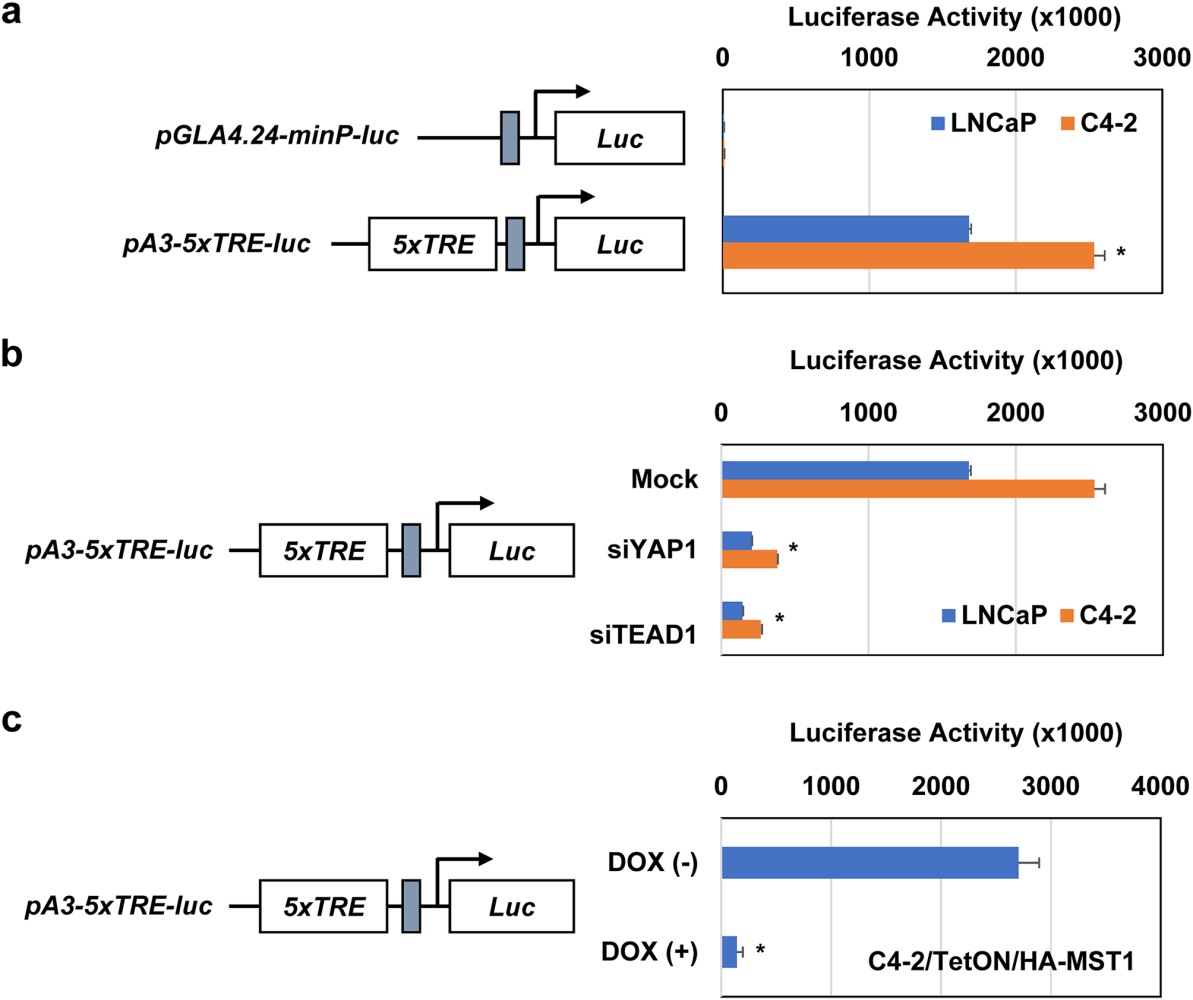
YAP1 and TEAD1 activate the EPHA3 promoter-directed reporter gene. (**a**) Luciferase activity of the pGLA4.24-minP-Luc (mock) or TEAD-responsive pA3-5xTRE-Luc (test) vector in LNCaP and C4-2 cells grown in serum fed-conditions. Schematic illustrations represent a pGLA4.24-minP-Luc and pA3-5xTRE-Luc vector. (**b**) Luciferase activity of pA3-5xTRE-Luc vector in LNCaP and C4-2 cells transiently transfected with scramble (mock) or with YAP1 or TEAD1 siRNA, *P < 0.001. LNCaP and C4-2 cells were transiently transfected with pA3-5xTRE-Luc plasmid DNA. (**c**) Luciferase activity of pA3-5xTRE-Luc vector in C4-2/TetON/MST1 cells exposed to Dox (−) or Dox (+). Relative luciferase activity was assessed at 48 h post-transfection and normalized to the total protein. Data are the representation of three independent experiments in triplicates.

The above observations indicate that YAP1 and TEAD1 bind the enhancer at TSS–3351 to drive EPHA3 expression. To test this possibility, we first fused the five copies of TREs from the TSS–3351 enhancer region to the pGL4.24-Min-luciferase (Luc) reporter vector, and the final product was designated pA3-5xTRE-Luc vector. Next, we evaluated the luciferase reporter activity of the control pGLA4.24-minP-Luc and the test pA3-5xTRE-Luc vectors in LNCaP and C4-2 cells. The results showed that 5xTRE from the TSS–3351 resulted in the luciferase reporter gene expression by thousands compared to the control vector, pGLA4.24-minP-Luc (Fig. 4a). Notably, C4-2 cells displayed significantly (*P* < 0.001) higher luciferase activity than LNCaP cells (Fig. 4a). To verify the specificity of the above observation, we then repeated the luciferase assay in the same cell lines after transiently co-transfect them with pA3-5xTRE-Luc vector and Mock, YAP1, or TEAD1 siRNA. Silencing YAP1 and TEAD1 suppressed the 5xTRE-directed reporter gene expression compared to the mock siRNA (Fig. 4b). In addition, induction of MST1 attenuated the TEAD-responsive 5xTRE-directed reporter gene expression compared with the control (Fig. 4c), which is reminiscent of the YAP1 and TEAD1 knockdown.

### TEAD1 regulates EPHA3 signaling

**Figure 5:**
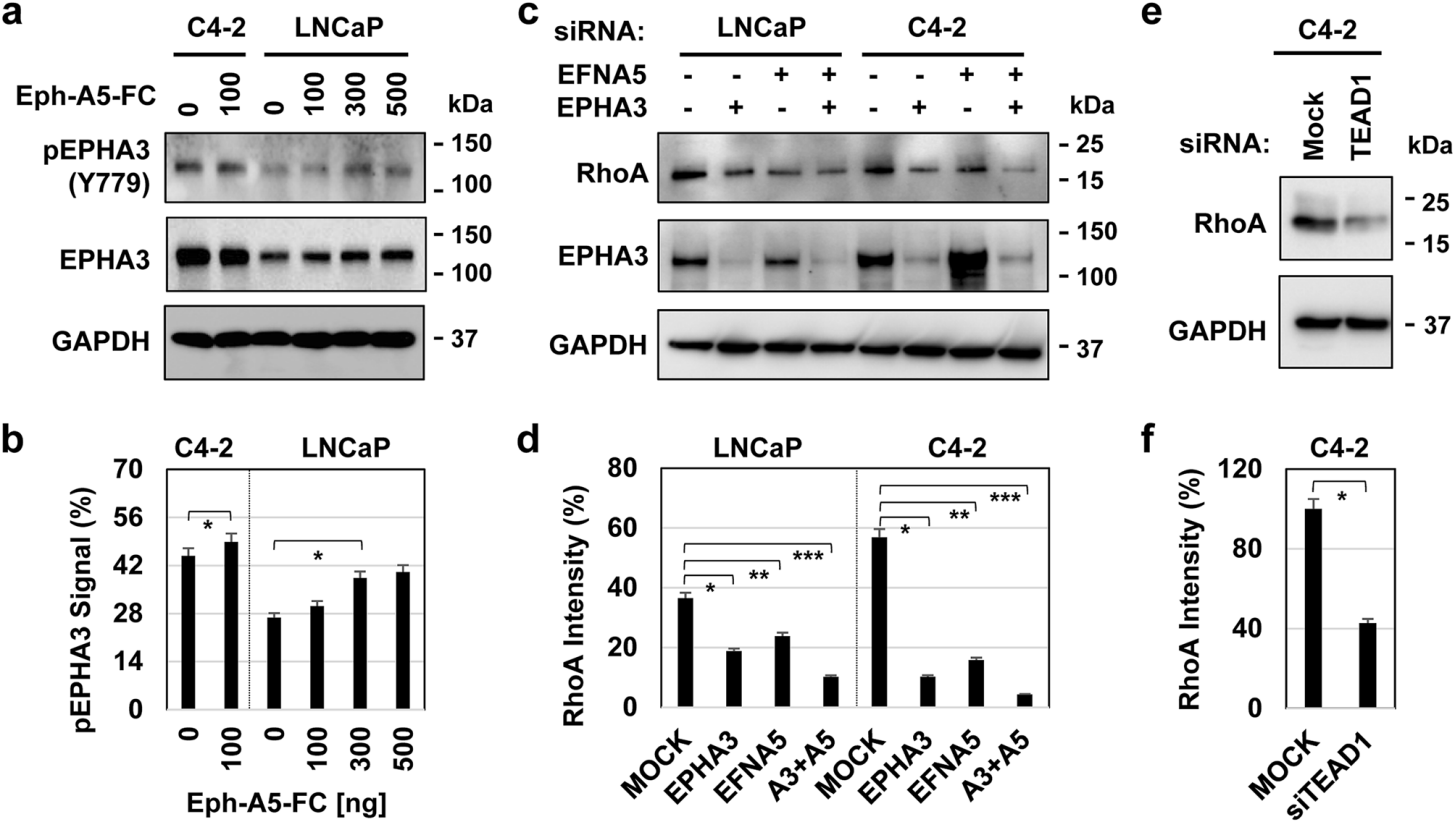
The activity of EPHA3 signals in LNCaP and C4-2 cells. (**a**) Analysis of phospho-Tyr779 and total EPHA3 proteins in LNCaP and C4-2 cells treated with ephrin-A5/Fc with varying doses in serum-depleted condition. Cells were serum-starved 24 h before being stimulated with Ephrin-A5/Fc, an activated Ephrin-A5 ligand. (**b**) Quantification of phospho-EPHA3-Tyr779 normalized to total EPHA3 and GAPDH protein levels; *P > 0.05 for C4-2 and *P < 0.01 for LNCaP cells. (**c**) Levels of the RhoA and EPHA3 proteins in LNCaP and C4-2 cells that were transiently transfected with mock (−), EPHA3 (+), or/and *EFNA5* (+ , Ephrin-A5 ligand) siRNA. (**d**) Quantification of the RhoA protein blot normalized to total GAPDH protein levels; *, **, ***P < 0.01 for both C4-2 and LNCaP cells. (**e**) Levels of RhoA and EPHA3 proteins in C4-2 cells were transiently transfected with mock (−) or TEAD1 (+) siRNA. Whole-cell lysates were prepared from cells grown in reduced-serum and analyzed at 48 h post-transfection. Membranes were probed with the protein-specific antibody. The GAPDH was used as an internal control in WB. (**f**) Quantification of the RhoA protein blot normalized to total GAPDH protein levels. ImageJ software was used to quantify the phospho-EPHA3 and total RhoA, EPHA3, and GAPDH proteins; *P < 0.01. Data are the representation of at least three independent experiments.

Ligand-mediated EphA receptor clustering is an essential mechanism for Eph receptor activation^56^. Also, evidence indicated that the receptor oligomerization or clustering resulted in phosphorylation of the tyrosine (Tyr) residues on EPHA3^57^. Phospho-Tyr779 in the EPHA3 kinase domain and phospho-Tyr596 and phospho-Tyr602 in the juxtamembrane are crucial for EPHA3 activation^57^. Therefore, we assessed the effects of dimeric ephrin-A5 ligand on phospho-EPHA3 protein in LNCaP and C4-2 cells. Although ephrin-A5-FC exposure induced phospho-Tyr779 in LNCaP cells in a dose-dependent manner, ephrin-A5-FC treatment did not change phospho-Tyr779 levels in C4-2 cells (Fig. 5a,b and Fig. S8a). However, it was apparent that phospho-Tyr779 levels at baseline were much higher in C4-2 than in LNCaP cells. This observation suggests that gene amplification results in constitutive activation of EPHA3, likely independently of the ephrin-A5 ligand.

In addition, a published study showed that EPHA3 could regulate cell motility by promoting RhoA expression^44^. To determine whether EPHA3 modulates RhoA expression, we transiently silenced ephrin-A5 and EPHA3 by siRNA in LNCaP and C4-2 cells, followed by western blotting. Knockdown of EPHA3 and ephrin-A5 markedly reduced the immunoreactivity of the RhoA protein compared to the control siRNA (Fig. 5c, d; Fig. S8b). Also, knockdown of TEAD1 significantly suppressed RhoA expression (Fig. 5e, f; Fig. S8c), indicating a functional link between YAP1/TEAD1 and EPHA3 signaling.

### EPHA3 silencing reduces cell–cell interaction and motility

**Figure 6:**
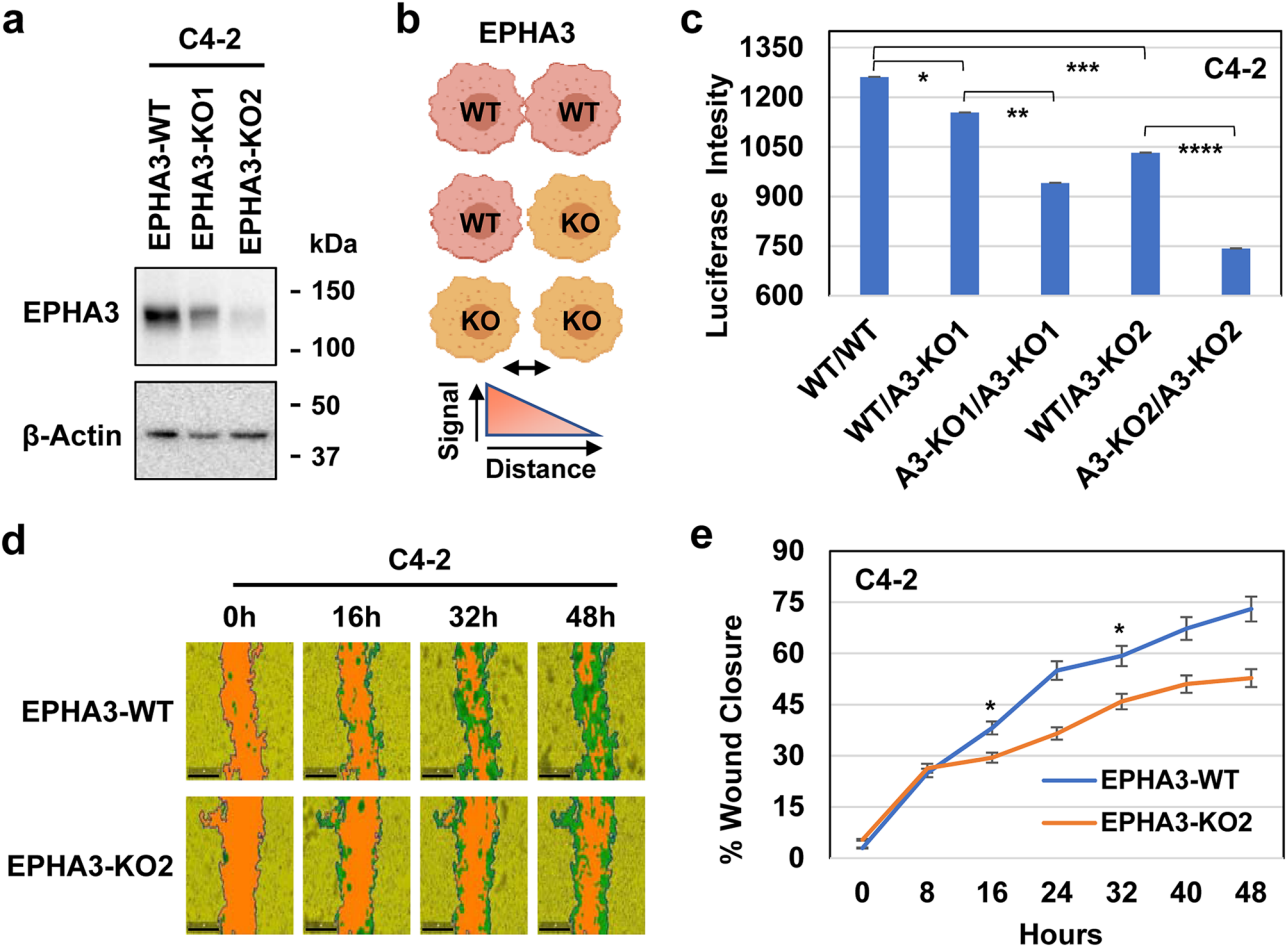
EPHA3 regulates cell–cell interaction and cell motility. (**a**) WB analysis of EPHA3 protein in EPHA3-KO C4-2 cell clones. The β-actin protein blot was used as an internal control. (**b**) Schematic representation of cell–cell interaction between EPHA3-WT/WT, EPHA3-WT/KO, or EPHA3-KO/KO cells. Light intensity inversely correlates with the distance between the two cell types. **(c**) Cell proximity assay (CPA) was conducted using EPHA3-WT and EPHA3-KO cell lines, as illustrated in panel b. *P < 0.03 and **, ***, ****P < 0.001*. *Data represent three independent experiments. The CPA system utilizes beta-galactosidase (β-gal) and luciferase (Luc) enzymes expressed by β-gal and Luc vectors. Bioluminescent signals are produced upon the addition of luciferase substrate, A3: EPHA3. (**d**) Micrographs are the representation of wound closure in select time, h: hour. Size bars: 20 µm. (**e**) The graph shows quantification of wound closure as a function of time, *P < 1.14E-13. Data represent from two independent experiments with eight data points.

YAP1 regulates cell contact and motility^58^, but the mechanism remains elusive. Therefore, we propose that EPHA3 is a critical mediator of cell contact and motility by YAP1^28^. To test this idea, we first generated the EPHA3-KO C4-2 cell lines (EPHA3-KO1 and EPHA3-KO2) using the CRISPR/Cas9 technology along with the EPHA3-WT control cell (Fig. 6a; Fig. S9). Then, we conducted a cell proximity assay to evaluate the effects of EPHA3 loss on cell–cell interaction in vitro. The cell proximity assay utilizes a bioluminescent-based luciferase assay system, and the intensity of the signal inversely correlates with the distance between cells (Fig. 6b). The cell proximity assay requires the luciferase and β-galactosidase reporter vector system. We transiently transfected individual reporter vector into the EPHA3-WT and EPHA3-KO cell lines. The cell carrying one kind of vector was mixed 1:1 ratio and then reseeded in a 96-well plate, followed by substrate addition and quantification of light intensity produced when the cells are in proximity (Fig. 6b). The combination of EPHA3-WT/KO and EPHA3-KO/KO significantly reduced cell–cell interaction compared to EPHA3-WT/WT (Fig. 6c). Notably, a complete loss of EPHA3 (EPHA3-KO2) attenuated cell–cell interaction more than a partial EPHA3 loss (EPHA3-KO1) (Fig. 6c). In addition, a scratch wound assay showed that EPHA3-KO2 significantly reduced wound closure as a function of time relative to EPHA3-WT (Fig. 6d,e). Altogether, EPHA3 is a key mediator of cell–cell interaction and cell motility downstream of the Hippo-YAP pathway.

## Discussion

We have identified the STK4/MST1-YAP1-TEAD1 axis as a crucial transcriptional regulator of EPHA3 expression and functions. Our study showed that YAP1, in concert with TEAD1, promoted EPHA3 expression. In addition, our chromatin immunoprecipitation and promoter-reporter assays established that YAP1 and TEAD1 enable EPHA3 promoter activation by binding to TREs, which likely serve as a putative distal enhancer. Furthermore, we demonstrated that EPHA3 gene silencing significantly reduced cell–cell interaction and motility. To our knowledge, this study is the first to show the molecular and functional links between the Hippo-YAP pathway and the ephrin family of receptor tyrosine kinases. This interaction is biologically relevant because both signaling mechanisms regulate similar cellular events, like differentiation, stem cell biology, developmental processes, and carcinogenesis.

Our study revealed that the YAP1 and TEAD1 proteins positively regulate EPHA3 expression. We computationally predicted the three putative TREs within the − 5 kb of the EPHA3 distal promoter relative to TSS^59^. The JASPAR^52^ and ConSite^53^ algorithms predicted that TRE at TSS–3551 is a putative binding site for TEAD1 and TEAD3 but not TEAD2 and TEAD4, suggesting that the TEAD family of transcription factors selectively regulate their target genes. Furthermore, our ChIP-qPCR assay verified that TEAD1 and YAP1 interacted with the genomic DNA fragments containing the TSS–3551, TSS–1841, and TSS–887 DNA sequence or TRE within the EPHA3 promoter. We identified the DNA sequence or TRE at TSS–3551 and TSS–1841, but not TSS–887, as enhancers for TEAD1. Our findings are consistent with the literature showing that YAP1 and TEAD1 primarily exert their transcriptional activity through distal enhancer^60,61^.

Moreover, one study suggested that AR could cooperate with the SP1 transcription factor to regulate EPHA3 expression in response to androgen hormone signaling^38^. In that study, the author identified an androgen response element (ARE) within the − 1 kb proximal promoter of the EPHA3 gene. These findings suggest that other transcription factors could also modulate EPHA3 expression, perhaps in a tissue-specific or context-dependent fashion. A published study indicated that YAP1 interacted with AR, which is critical for AR-dependent gene transcription and functions^19^; however, whether YAP1 and TEAD1 collaborate with AR to regulate EPHA3 expression warrants further investigation, which is not the subject of the current study.

Furthermore, Hippo signaling controls cell–cell interaction and cell migration, likely modulating actin polymerization and cytoskeletal dynamics^62,63^. YAP1 regulates focal adhesion and cytoskeleton stability^58,64,65^, probably intersecting with RhoA signaling^66^. Also, EPHA3 is known to control cell contact, focal adhesion, and cell rounding^42^. Evidence suggested that the CrkII adaptor protein and RhoA could function as critical intermediates between EPHA3 and the actin cytoskeleton^44^. Our study showed that silencing EPHA3 and ephrin-A5 diminished RhoA expression significantly. Our analysis also demonstrated that TEAD1, a key mediator of YAP1 transcriptional activity, significantly reduced RhoA protein levels, which coincided with the loss of EPHA3 expression. Furthermore, EPHA3-KO dramatically decreased cell–cell interaction and cell motility compared to EPHA3-WT. Therefore, our findings suggest that EPHA3 is critical for the regulation of cell-contact and cell motility by the Hippo pathway, demonstrating a functional link between Hippo and EPHA3 signaling.

The molecular mechanisms that modulate Eph receptor activation are complex. The receptor clustering, dimerization, and autophosphorylation are critical for activating Eph receptor signaling^42^. Evidence suggests that phospho-Tyr779, -Tyr596, and -Tyr602 activate EPHA3 through receptor clustering^57,67^. Our data in the current study showed that ephrin-A5 was necessary for EPHA3 activation in the androgen-dependent LNCaP cell line, as assessed by phospho-Tyr779, a critical activator for EPHA3. However, the C4-2 cell line, which expressed the high levels of EPHA3 protein, coinciding with enhanced phospho-Tyr779, did not respond to exogenous ephrin-A5 signaling. One plausible explanation is that amplification may lead to EPHA3 activation independently of its ligand. This interpretation is consistent with the literature showing the ligand-independent activation of other membrane-associated receptor tyrosine kinases. For example, the EGFR-WT tyrosine kinase does not require a ligand when amplified^68,69^. Thus, it appears that EGFR-WT resulted in different and mutually exclusive outputs depending on the presence of its ligand. In addition, FGFR1 amplification resulted in ligand-independent activation^70^. Likewise, insulin-like growth factor receptor-1 (IGF-1R) resulted in the ligand-independent activation of the c-MET receptor tyrosine kinase^71^. Taken together, the role of constitutively active EPHA3 signaling deserves further investigation, perhaps in the context of other membrane-associated receptor tyrosine kinases.

Furthermore, EPHA3 plays a significant role in the biology of various hematological and solid cancers^72^. EPHA3 was discovered initially in a pre-B acute lymphoblastic leukemia cell line^73^. In addition, upregulation of EPHA3 has been reported in sarcomas, lung cancer, melanoma, and glioblastoma^74^. EPHA3 has abundantly expressed in glioma tumor-initiating cells and plays a crucial role in keeping tumor cells in a less differentiated state, likely altering mitogen-activated protein kinase signaling^46^. Published research suggested a link between increased EPHA3 expression and tumor depth, stage, and metastasis in gastric cancer^75^. Also, upregulation of EPHA3 was observed in the AR-positive LNCaP and 22Rv1 cell lines^38,76^. Here, we showed that prostate cancer cell lines expressed the differential levels of EphA receptors and their ligands. In addition, we noted that, unlike AR-negative cells, AR-positive cells confer high levels of EPHA3 and ephrin-A5. Markedly, the levels of EPHA3 in the metastatic castration-resistant prostate cancer cell line, C4-2, were much higher than its less aggressive, parental LNCaP cell counter. Collectively, EPHA3 may have a critical role in advancing human prostate cancer. However, whether EPHA3 promotes or suppresses prostate cancer progression requires further investigation^77^.

**Figure 7:**
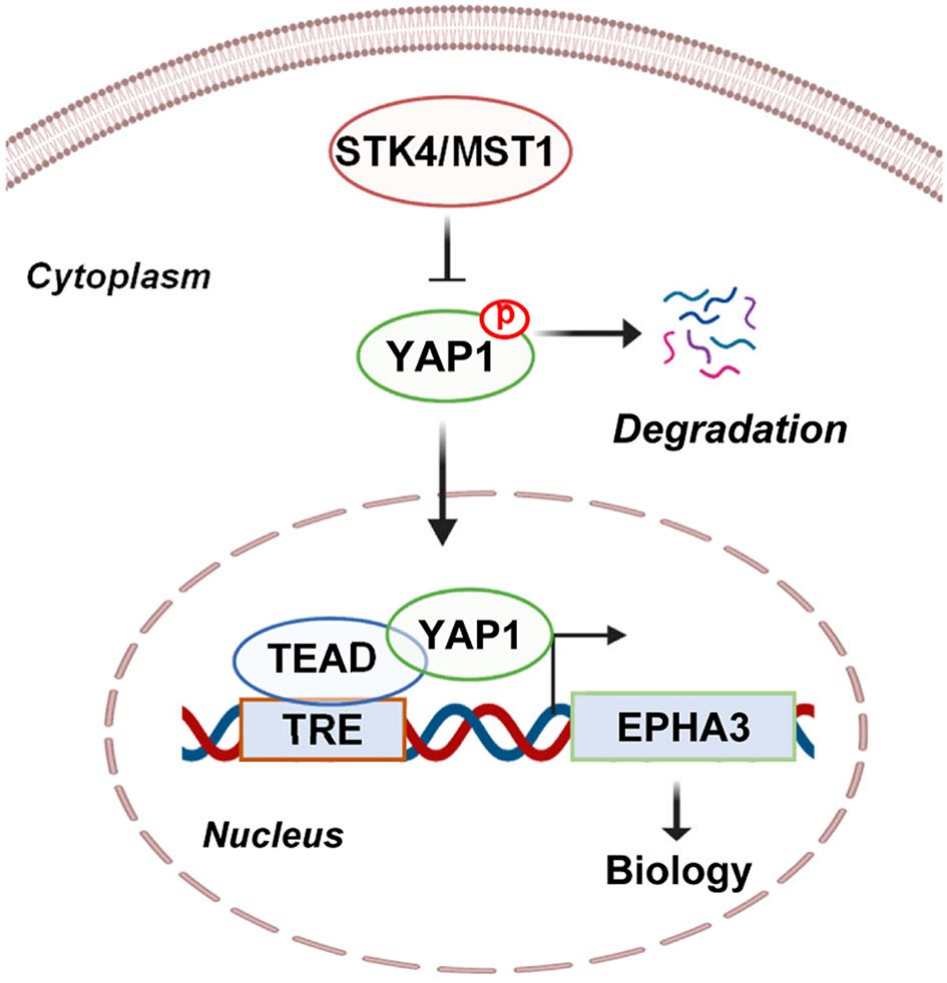
The model summarizes the results of the study. The YAP1 and TEAD1 proteins bind the TRE and transcriptionally regulate the EPHA3 expression and biology downstream of the Hippo pathway. The membrane and nuclear boundaries and double-stranded DNA images were created using the http://www.BioRender.com web page, and the remaining content was constructed using Microsoft PowerPoint.

In summary, our current study revealed novel mechanistic and functional links between the Hippo-YAP pathway and the EPHA3 receptor. Moreover, our study identified YAP1 and TEAD1 as potent activators of EPHA3 and biology (Fig. 7). Both YAP1 and EPHA3 modulate cell–cell interaction and cell motility. Thus, when combined with future studies, the result from this investigation will help us better understand the cellular biology relevant to the developmental processes and mechanisms of human diseases such as cancer.

## Methods

### Cell lines

LNCaP, C4-2, and 22Rv1 cell lines were purchased from American Type Culture Collection (ATCC). In addition, the PC3 cell line was available in the laboratory. Cells were grown in RPMI 1640 cell culture medium supplemented with 10% fetal bovine serum (FBS) and 1% penicillin and streptomycin antibiotics at 37 °C in 5% CO_2_, humidified-cell culture incubator.

### RNA extraction and cDNA synthesis

According to the manufacturer's instruction, total RNA from cells at 60–70% confluence was isolated using TRIzol RNA isolation reagent (Invitrogen, 15,596,026). For cDNA synthesis, 1 μg of total RNA was reverse transcribed in a reaction containing 0.5 μg random hexamers, GoScript Reverse Transcriptase, 1 × GoScript reaction buffer, 1 mM dNTPs, and 5 mM MgCl2, as instructed (Promega, A5000). The reaction conditions were 5 min at 25 °C for annealing and 40 min at 42 °C for extensions using C100 Touch thermal cycler (Bio-Rad Laboratories).

### Polymerase chain reaction

Semi-quantitative reverse transcriptase (RT)-polymerase chain reaction (PCR) was conducted using GoTaq green master mix (Promega, M7822) and 2 μl cDNA. RT-PCR was carried out using C100 Touch thermal cycler (Bio-Rad laboratories). The RT-PCR program was: 2 min at 95 °C for initial denaturation, 45 s at 95 °C for denaturation, 60 s at 65 °C for annealing, 3 min at 72 °C for an extension for 30 cycles, and 5 min at 72 °C for a final extension. Quantitative-PCR (qPCR) was performed using GoTaq 1-Step RT-qPCR System (Promega, A6020). 100 ng total RNA and 100 nM ephrin primers were used in qRT-PCR. The 1-Step RT-qPCR program was: 15 min at 37 °C for reverse transcription, 10 min at 95 °C for the inactivation of RT reaction, 10 s at 95 °C for denaturation, followed by 30 s at 60 °C for annealing, and 30 s at 72 °C for an extension for 40 cycles. The levels of EPHA3 transcripts were determined using a 2-ΔCt method and normalized to the 18S ribosomal RNA. 18S ribosomal RNA was used as an internal control in PCR. Primers used in PCR are listed in Table 1. qPCR was carried out using the CFX Connect Real-Time PCR Detection System (Bio-Rad Laboratories).

**Table 1:** Primer sets that were used to evaluate the expression of EphA and EphB receptors and their ligands.

Primer ID	Forward Sequence (5ʹ to 3 ʹ)	Reverse Sequence (5ʹ to 3ʹ)
EPHA1	GAAAGAACCGAGGCAACTAGAG	CC ATC TGGTAC C GTTC TTC ATC
EPHA2	GTCCCTCTAGTGCCTTCTTTAG	CCTCAACACAACCAAGCATC
EPHA3	CTCCATCTCTGGTGAAAGTAGC	CC AC AGAAC C TC C C AATC A A
EPHA4	C GTTC AC AC TTC GC TC C TTTA	TCTTCCACGGGCTTTGTAATC
EPHA5	GTCCCAATGGAATCATCCTAGAG	TTCAAGCCCTCTGCAGTAATAG
EPHA6	A AG AGC GTG AC AC TC C TA A AC	CATGGCAAGAACCCTCAA TTTC
EPHA7	GTGGGCATCCACAAACAAAC	C AG AG C C C AC TAC AG AG AAATG
EPHA8	GGGTGTTCTCACAAGGTCAT	CGGATAAGCACACGCTCATA
EFNA1	C TTAAAGAGGG AC AGGC TG AAG	GGCTGCTAGGTGATAGCTTATG
EFNA2	GGGAACCTCTTGGCGATTT	GCTACAACGGCAGGGAATAA
EFNA3	TTCTCTCTGGGCTACGAGTT	CACCTTCATCCTCAGACACTTC
EFNA4	C C C TC ATC AC AGGCTAAAGAAG	TACCAAATCCCAGTCCTCC
EFNA5	GAACACCAGAGATCCACCTAAC	GGGAGGCAGGAACAAGTTTA
EPHB1	GTAGCAGGAAACGGGCTTATAG	GTTGGGATCCTCGTAAGTGAAG
EPHB2	GGATGTACCCATCAAGCTCTAC	CAGACGGTGCCATTCTCAA
EPHB3	GAGAAGCTGCAGCAGTACAT	GTCGATCTCCTTGGCAAACT
EPHB4	GGAGGGAAC CTGTTTC ACTATG	GATGACCAAGGCACTGTTCT
EPHB6	ATGATCCGCAAGCCAGATAC	GGTGAGTCCAGACAAGGAAAG
EFNB1	TCCCTCACTCTCACGGTAAT	AGC ICTGCAGGGCAAA! AG
EFNB2	CCAAAGTGCGTGTGTGTATG	GGCAACCCTCCACAGAAATA
EFNB3	CTTTCCCTCTCTTCCGTCTCTA	ATGGGAGAGGCACAGGATAA

### Protein analysis

Total proteins from monolayer cells were isolated using lysis buffer (20 mM HEPES, pH 7.4, 150 mM NaCl, 0.5% NP-40, 1 mM EDTA, 1X protease inhibitors, and phosphatase inhibitors cocktails (Calbiochem, #539131)) as previously described^19,23^. Cells were briefly washed with ice-cold phosphate buffer saline (PBS) three times before cell lysis. The lysate was incubated for 40 min on ice and centrifuged at maximum speed for 15 min at 4 °C. Protein concentrations were measured using Pierce rapid gold BCA protein assay kit as instructed by the manufacturer (ThermoFisher Scientific, A53227), and the concentrations were measured using BioTek Synergy H1 spectrometer. Proteins were separated by 8% SDS–PAGE and transferred to nitrocellulose membrane (Bio-Rad Laboratories, 1620112). Membranes were incubated with blocking buffer (0.1% Tween-20 and 5% (w/v) non-fat milk prepared in PBS) for 1 h at room temperature and then washed three times, 5 min each, with washing buffer (PBS-T (0.1% Tween-20)). The membranes were probed with the primary antibody to EPHA3/A4/A5 (Cell Signaling Technology (CST, #8793, 1:1000), SCBT (sc-514209, 1:100), phospho-EPHA3 (Tyr779) (CST, #8862, 1:1000), YAP1 (CST, #8418 and 12395S, 1:1000), TEAD1 (CST, #12292, 1:1000), TEAD3 (CST, #13224, 1:1000), RhoA (CST, #2117, 1:1000), HA-Tag (CST, #3724, 1:1000), beta-actin (Sigma-Aldrich, A2228, 1:3000), or GAPDH (CST, #5174, 1:2000) at 4 °C overnight, followed by three washes 5 min each. Then, the membranes were incubated with the secondary antibody linked to the HRP anti-rabbit or anti-mouse IgG (CST, #7074 and 7076, respectively, 1:2000) for 1 h at room temperature and then washed three times, 5 min each. The protein signal was visualized using a Luminata Forte Western HRP substrate (Millipore Sigma, WBLUFO500) and ChemiDoc MP Imaging (Bio-Rad, #12003154). Western blots were processed and constructed using Photoshop (brightness, contrast, and cropping only) and PowerPoint applications. ImageJ was used to quantify protein signals in western blot images. Full-length blots were included in the manuscript as Supplementary Figures in the Supplementary Information. Here, we want to state that it is difficult to capture the boundaries of the membrane because images were not captured using X-ray film. Micrographs provided were digitally captured using the ChemiDoc MP Imaging system from Bio-Rad Laboratories.

### Immunofluorescence and microscopy

2 × 10^4^ cells seeded in glass-bottom chamber slides. Cells were fixed with 4% paraformaldehyde (PFA) for 20 min, permeabilized with 0.3% Triton-X-100 for 5 min and blocked with blocking buffer (2% BSA (bovine serum albumin) with 0.3% Triton-X-100 prepared in PBS) for 1 h at room temperature. Then, cells were incubated with the EPHA3 primary antibody conjugated to Alexa Fluor 488 (SCBT, sc-514209 AF488, 1:50) at 4 °C overnight. Cells were washed three times with PBS and mounted using Prolong Gold-Antifade reagent with DAPI (CST, #89615). EPHA3 protein signal was captured using confocal microscope (Zeiss, LSM 700, Confocal Microscope) at 40X or 63X magnification.

### RNAi transfection

On-TARGETplus non-targeting Mock control (D-001810-01-05) and ON-TARGETplus SMARTpool siRNA reagent targeting human EPHA3 (L-003117-00-0005), EFNA5 (L-011649-00-0005), YAP1 (L-012200-00-0005), and TEAD1 (L-012603-00-0005) were purchased from Dharmacon (Horizon Discovery, Inc.). Table 2 shows the pool of gene specific siRNA sequences used in RNAi transfection experiments. Briefly, 2 × 10^5^ cells were plated in a six-well plate overnight. Cells at 50–60% confluence were subjected to transfection. 50 nM of the gene-specific siRNA were transfected with DharmaFect-3 transfection reagent according to the manufacturer's instruction (Dharmacon-Horizon, T-2003-01) in Opti-MEM reduced serum medium (Life Technologies). Western blotting was conducted at 72 h post-transfection to assay EPHA3, YAP1, and TEAD1 expression.

**Table 2:** Sequences of non-targeting mock control and SMARTpool siRNA targeting human EPHA3, EFNA5, YAP1, and TEAD1 genes.

Gene ID	On-Target siRNA Sequence (5ʹ to 3ʹ)
MOCK Control	UGGUUUACAUGUCGACUAA
EPHA3	CCUCAAGCCUGACACUAUA GUUAGAGGGUCUUGUGUCA ACAAGGCAUUGGAUGGUAA GGUGAAAUUUCGAGAGCAU
EFNA5	GAAGAAGGUCCUGUCUAAA GAAUGUAACCGGCCUCACU CGAGAACGCGGCACAAACA CAAAUGGACCGCUGAAGUU
YAP1	GCACCUAUCACUCUCGAGA UGAGAACAAUGACGACCAA GGUCAGAGAUACUUCUUAA CCACCAAGCUAGAUAAAGA
TEAD1	CGAUUUGUAUACCGAAUAA CACAAGACGUCAAGCCUUU AAACAGGGAUACACAAGAA GAAAGGUGGCUUAAAGGAA

### EPHA3 knockout cell lines

C4-2 cells were seeded in a six-well plate (2 × 10^5^ cells per well) overnight before transfection. Using FuGene HD transfection reagent (Promega, E2311), cells were transfected with 1–3 μg human EPHA3 HDR plasmid (Santa Cruz Biotechnology (SCBT, sc-401565-HDR) and EPHA3 CRISPR/Cas9 plasmid(h) (SCBT, sc-401565) for 72 h, followed by puromycin (Pur) treatment (2 μg/mL) to select resistant cell clones. Individual clones were transferred to a new tissue culture plate and grown in a complete medium supplemented with puromycin. Western blot and quantitative PCR were performed to verify the loss of EPHA3 expression.

### TRE identification

The EPHA3 promoter region was analyzed using the EPD web portal (https://epd.epfl.ch/)^51^ to search for potential TEAD Responsive Elements (TREs) or the TEAD cis-acting DNA. EPHA3 promoter corresponding to − 5000 bp to + 100 bp DNA sequence relative to transcription starting site (TSS) were scanned. Based on the stringent p-values, three putative TREs were located at TSS–3551 (TACATTCCACGT) with p-value (*P* < 0.00001), at TSS–1841 (CAGATTCCTGGG) with p-value (*P* < 0.0001), and TSS–887 (GATTTGGAATGTT) with p-value (*P* < 0.0001). TEAD1 and TEAD3 occupied the TSS–3551 with a cut-off *P* < 0.00001 value. Then, we used the JASPAR transcription factor binding site database (http://jaspar.genereg.net/)^52^ and ConSite (http://consite.genereg.net/)^53^ to verify the putative TREs. The identification of TREs was detailed in Supplementary Information 2.

### Chromatin immunoprecipitation

According to the manufacturer's instruction, the chromatin immunoprecipitation (ChIP) assay was performed using Magna ChIP™ HiSens ChIP Kit (Millipore, Sigma, #17–10460). Briefly, LNCaP and C4-2 cells were plated in a complete RPMI-1640 medium, and cells at 80% confluence were cross-linked by formaldehyde (1% to final concentration) at room temperature for 10 min. The cross-linking was quenched with 0.125 M glycine for 5 min at room temperature. Cells were collected in PBS containing 1 × protease inhibitor and centrifuged. After decanting the supernatant, the cell pellet was washed with ice-cold PBS and lysed in 600 µl of nuclei isolation buffer. The chromatin DNA was sheared in a water bath at 4 °C using Covaris S2 sonicator in a microTUBE AFA Fiber Snap (Covaris, #520077). The maximum sample volume was 130 µl. The sonication parameters are as follows: duty cycle 10%, intensity 4, cycles/burst 200, 15 cycles for 30 s each, with at least 30 s of cooling between cycles. The sonicated lysates were centrifuged at 4 °C for 15 min with maximum speed, and the supernatant was collected, aliquoted, and stored at − 80 °C until analysis. For the ChIP assay, 10 µl of magnetic beads were incubated with an appropriate concentration of IgG (negative control), YAP1, TEAD1, or H3K27ac antibody (test) in ~ 190 µl sonication/ChIP/wash (SCW) buffer at 4 °C overnight. The next day, the magnetic bead-antibody complexes were washed two times with SCW buffer. Then, 5 µl of protein-bound chromatin DNA fragments were added to the antibody-magnetic bead complexes and resuspended in 500 µl in SCW buffer, followed by incubation at 4 °C overnight with gentle rotation. The beads were collected and washed with the SCW and low stringency IP buffer. The antibody-protein-crosslinked DNA and the input protein-crosslinked DNA samples were reversed by proteinase K digestion at 65 °C for 2 h in 50 µl ChIP elution buffer. Purified DNA fragments were analyzed by quantitative PCR (qPCR) using SYBER green master mix (Applied Biosystems, A46012) using a primer set designed to amplify the ChIP-DNA fragment, consisting of the predicted TREs in the EPHA3 promoter DNA regions: TSS–3551 bp, TSS–1841 bp, and TSS–887 bp, relative to the transcriptional start site (TSS). Quantitative PCR was performed according to the manufacturer's parameters: 10 min at 94 °C for initial denaturation, 20 s at 94 °C for denaturation, 1 min at 60 °C for annealing, and 30 s at 60 °C for extensions for 50 cycles. Table 3 shows the primer sets used in ChIP-qPCR reactions. The relative occupancy of TSS–887, TSS–1841, and TSS–3551 was normalized to the IgG signal, and the data were presented as fold enrichment.

**Table 3:** Primer sets were used in the ChlP-qPCR reactions to amplify the DNA fragments surrounding TREs with the EPHA3 promoter.

Primer ID	Forward Sequence (5ʹ to 3ʹ)	Reverse Sequence (5ʹ to 3ʹ)
EPHA3 TSS-3551	TTTACCCTCTGCTTTCCCTCAG	AGGAGTAAGAGAGGTTTGAGGA
EPHA3 TSS-1841	C C AGGGACC C C AAGGATTAC	CCACCACAGACTTACTGGACC
EPHA3 TSS-887	ATAAGTGAGAAAGTACGAGGACAT	TTCAACTTTCAACCACTGATAGTC

### Plasmid construction

Plasmid construction was performed as previously described^78^. Briefly, five copies of tandem TRE (5xTRE) from the EPHA3 promoter DNA region (TSS -3551) with the added restriction enzymes (RE) *NheI* (5' end)/*HindIII* (3' end) were obtained from IDT, Inc, followed by DNA annealing. DNA annealing was conducted using the duplex buffer (100 mM Sodium Acetate; 30 mM HEPES, pH 7.5) by heating the tubes at 94 °C for 2 min and gradually cooling at room temperature. The 5xTRE duplex oligo and pGL4.24[luc2P/minP] plasmid DNA were double digested with *NheI/HindIII* REs and separated on low melting agarose gel (OmniPur Agarose, Millipore, 2070), followed by gel purification using QIAprep Spin Miniprep Kit (Qiagen, #27104). Then, the 5xTRE DNA oligo was ligated upstream of the minimal promoter (minP) of pGL4.24[luc2P/minP] plasmid with 1:3 molar concentration. pGL4.24[luc2P/minP] plasmid (Promega, #8421) was purchased from Promega, Inc. T4 DNA Ligase (New England Biolabs, M0202S) was used in the ligation reaction. The final product, named as a pA3-5xTRE-Luc vector, was transformed into the DH5α competent cells according to the manufacturer's instruction (Invitrogen, #18265017). The positive colonies were picked up and amplified overnight at 37 °C with 225 rpm speed, then plasmid purification using a plasmid miniprep kit (Qiagen, #27104). The presence of the 5xTRE DNA insert was confirmed by *NheI/HindIII* double digestion, and PCR amplification using a primer set designed to prime the upstream and downstream of multiple cloning sites of the pGL4.24[luc2P/minP] vector. Plasmids generated through this study will be made available to other researchers upon request after publication. Table 4 shows 5' to 3′ 5xTRE single-stranded DNA sequences and primer sets used to verify the presence of the 5xTRE DNA insert.

**Table 4:** Oligonucleotides carrying the five copies of TRE from the TSS -3551 DNA region of the EPHA3 distal promoter.

Primer ID	Forward Sequence (5ʹ to 3ʹ)	Reverse Sequence (5ʹ to 3ʹ)
pA3-5xTRE-Luc	GCGCTAGCTACATTCCACGTGCGTACATTCCACGTGCGTACATTCCACGTGCGTACATTCCACGTGCGTACATTCCACGTGCGTACATTCCACGTGCGTACATTCCACGTGCGTACATTCCACGTAAGCTTGC	GCAAGCTTACGTGGAATGTACGCACGTGGAATGTACGCACGTGGAATGTACGCACGTGGAATGTACGCACGTGGAATGTACGCACGTGGAATGTACGCACGTGGAATGTACGCACGTGGAATGTAGCTAGCGC
pGL4.24-Luc-Seq	TAGCAAAATAGGCTGTCCC	TACCAACAGTACCGGATTGC

### Luciferase reporter assay

Luciferase reporter assay was conducted according to the manufacturer's instruction (Promega, E397A). Briefly, LNCaP, C4-2, and C4-2/TetON/HA-MST1 cells were seeded in a 12-well tissue culture plate. The next day, cells at 60–70% confluency were transiently transfected with mock (pGL4.24/luc2P-minP) or (pA3-5xTRE-Luc) luciferase reporter plasmid (1 µg DNA per well) using FuGene HD transfection reagent as instructed (Promega, E2311). 24 h post-transfection, C4-2/TetON/HA-MST1 were treated with (+) or without (−) doxycycline (Dox, 2 µ/ml) in TetON-approved serum-fed condition as described previously^79^. Luciferase reporter gene activity was determined at 72 h post-transfection. LUMIstar OPTIMA Microplate Luminometer (BMG LabTech) was used to measure Relative Luciferase Units (RLUs) in a 96-well plate (Dynex) with a 2-s measurement delay, followed by a 10-s measurement. RLUs were normalized to total protein, and the data were presented as luciferase activity.

### Cell proximity assay

Cell proximity reporter assay was performed according to the manufacturer's instruction (Abcam, ab253406). Briefly, wild-type and genetically modified C4-2 cells were seeded in a 6-well plate (2 × 10^5^ cells per well) overnight before transfection. First, cells were separately transfected with 1 μg of red luciferase reporter vector or β-galactosidase vector using FuGene HD transfection reagent (Promega, E2311), followed by incubation overnight. Then, 1 × 10^4^ cells from the red luciferase-transfected and β-galactosidase transfected well were mixed (1:1 ratio) and reseeded in a 96-well plate to a final density of 2 × 10^4^ cells/well, followed by incubation overnight. Next, the media were removed and replaced with 50 µl fresh medium 1 h before adding the substrate. Then, 50 µl of the substrate was added to each well and incubated for additional 45–60 min, and the luminescence intensity was measured using LUMIstar OPTIMA Microplate Luminometer.

### Wound healing assay

EPHA3-WT and EPHA3-KO2 cells were seeded and grown under steady-state conditions. At 24 h, monolayer cells were scratched using the p200 tip, and wound closures were measured for 48 h using the MuviCyte live-cell imaging system (PerkinElmer). The cell-free surface was measured at 0, 8, 16, 24, 32, 40, and 48 h. The results were presented as a percentage of wound closure.

### Statistics

A *t*-test were performed to determine the statistical significance between control and test samples. The *t*-test was performed using Microsoft Excel. Prior to t-test, we performed Q-Q plot to test the normality of our data (Fig. S10 and S11). A *p*-value equal to or less than 0.05 was considered significant.

## Supplementary Information

Supplementary Information 1.

Supplementary Information 2.
