# Supplementary material for: The Hippo effector YAP1/TEAD1 regulates EPHA3 expression to control cell contact and motility
Source: Sci Rep. 2022 Mar 9;12:3840. doi: 10.1038/s41598-022-07790-4 (PMC8907295; doi:10.1038/s41598-022-07790-4)
Supplement: Supplementary file 1 — Supplementary Information 1. [file 41598_2022_7790_MOESM1_ESM.pdf]

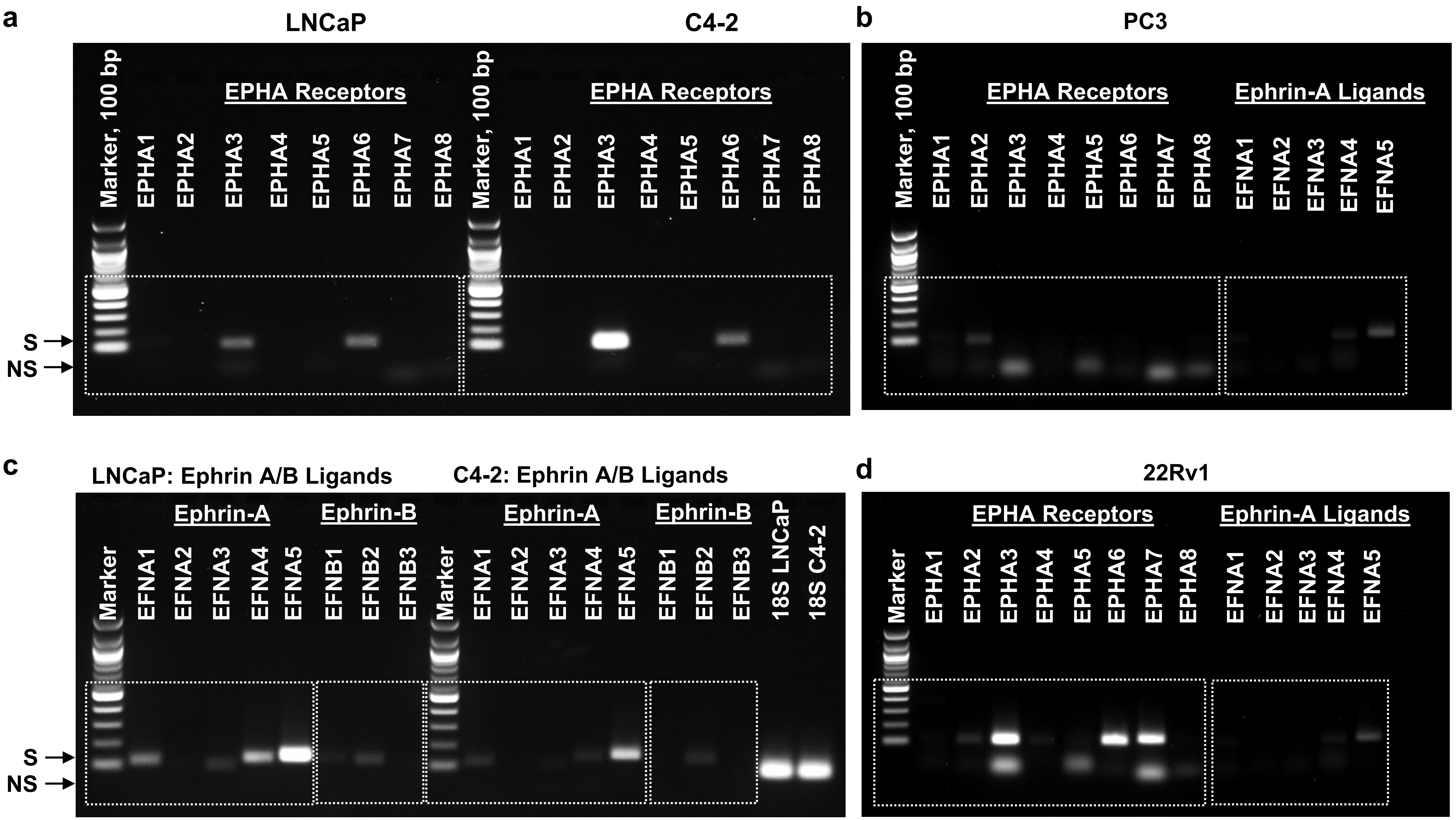

Figure S1. Images of full-length agarose gel (a-d). Gels with a dotted box were cropped to generate Figure 1a. S: Specific RT-PCR signal around 100 bp (the lowest size marker); NS: Non-specific RT-PCR signal. Size marker is 100 base pair (bp). Micrographs provided were digitally captured.

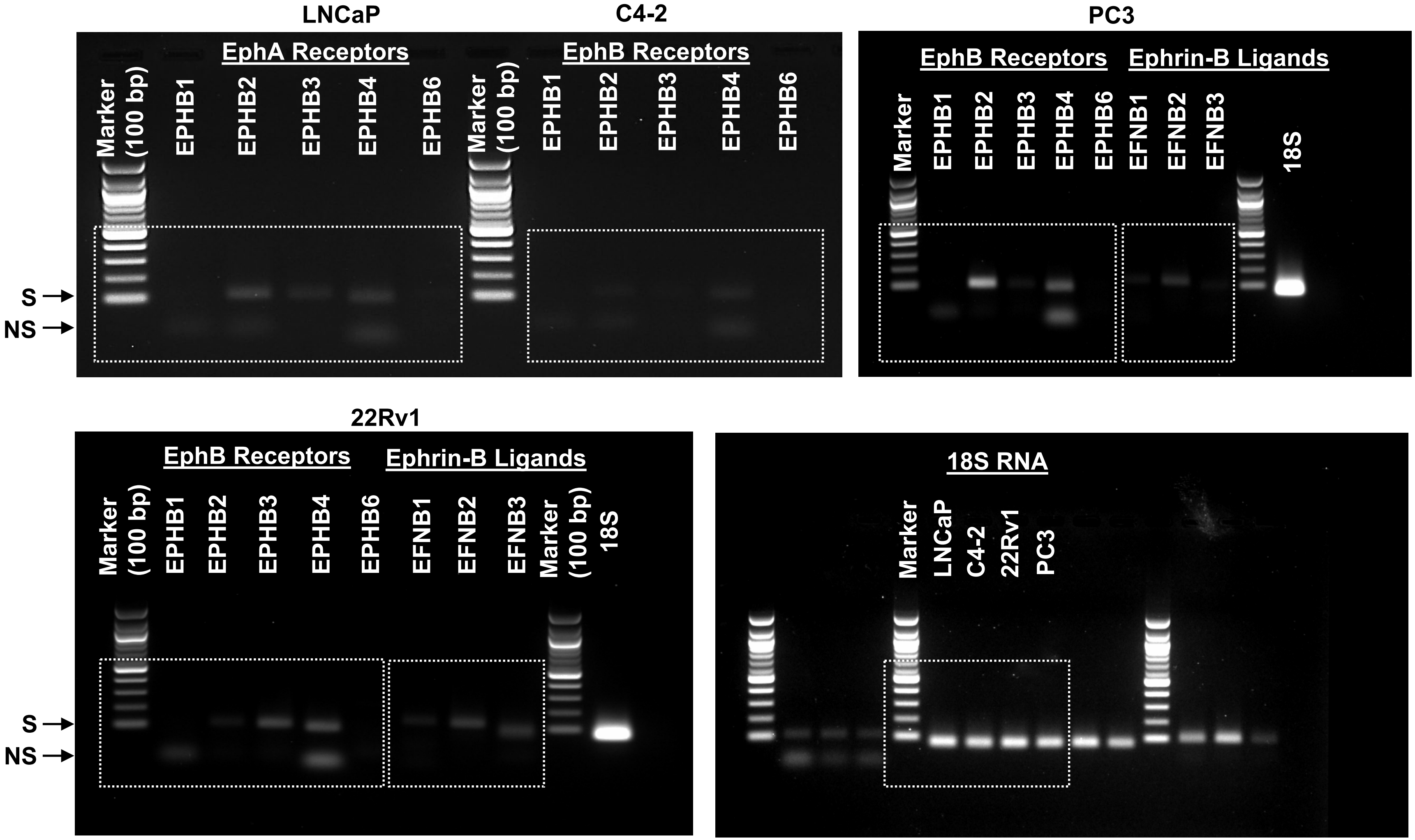

Figure S2. Images of full-length agarose gel. Gels with a dotted box were cropped to generate Figure 2b and 2c. S: Specific RT-PCR signal around 100 bp (the lowest size marker); NS: Non-specific RT-PCR signal. Size marker is 100 bp. Micrographs provided were digitally captured.

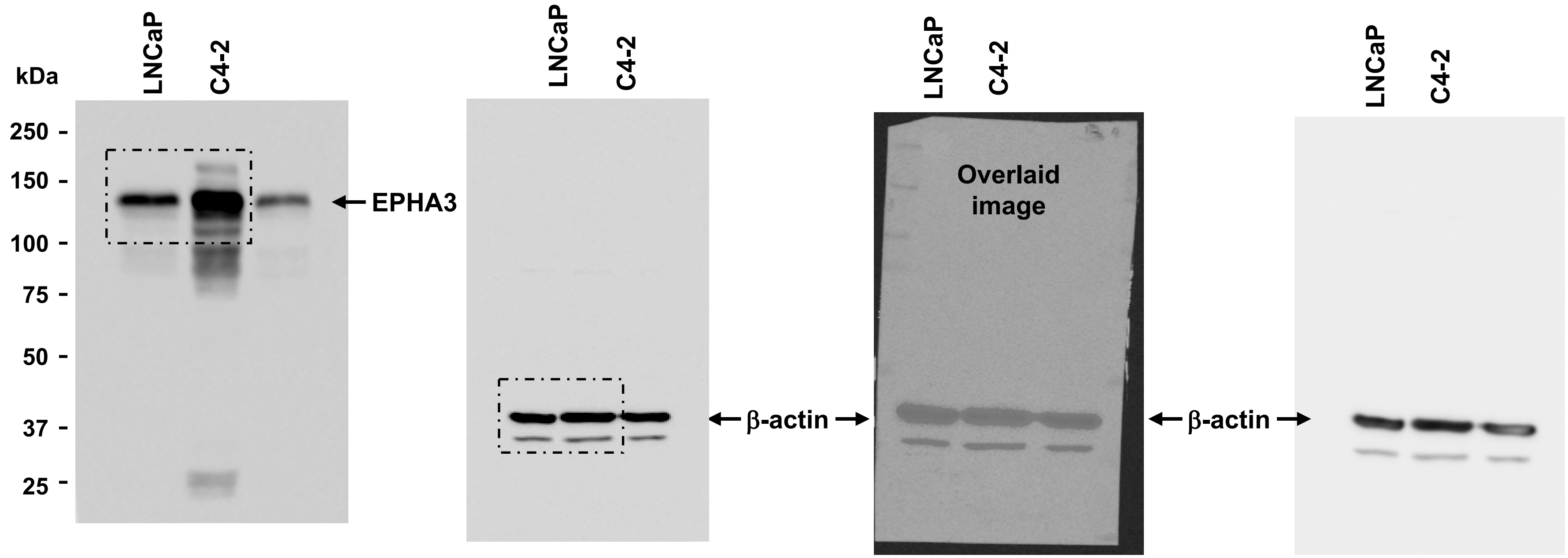

Figure S3. Images of full-length blots. The blots with a dotted box were cropped to generate figure 1e in the main manuscript. β-actin blot shows the different versions of exposure without rotation of the blots. Note that it is difficult to capture the boundaries of the membrane because images were not captured using X-ray films. Micrographs provided were digitally captured. The overlaid image was different exposure that shows the edges of the membrane with the corresponding blot.

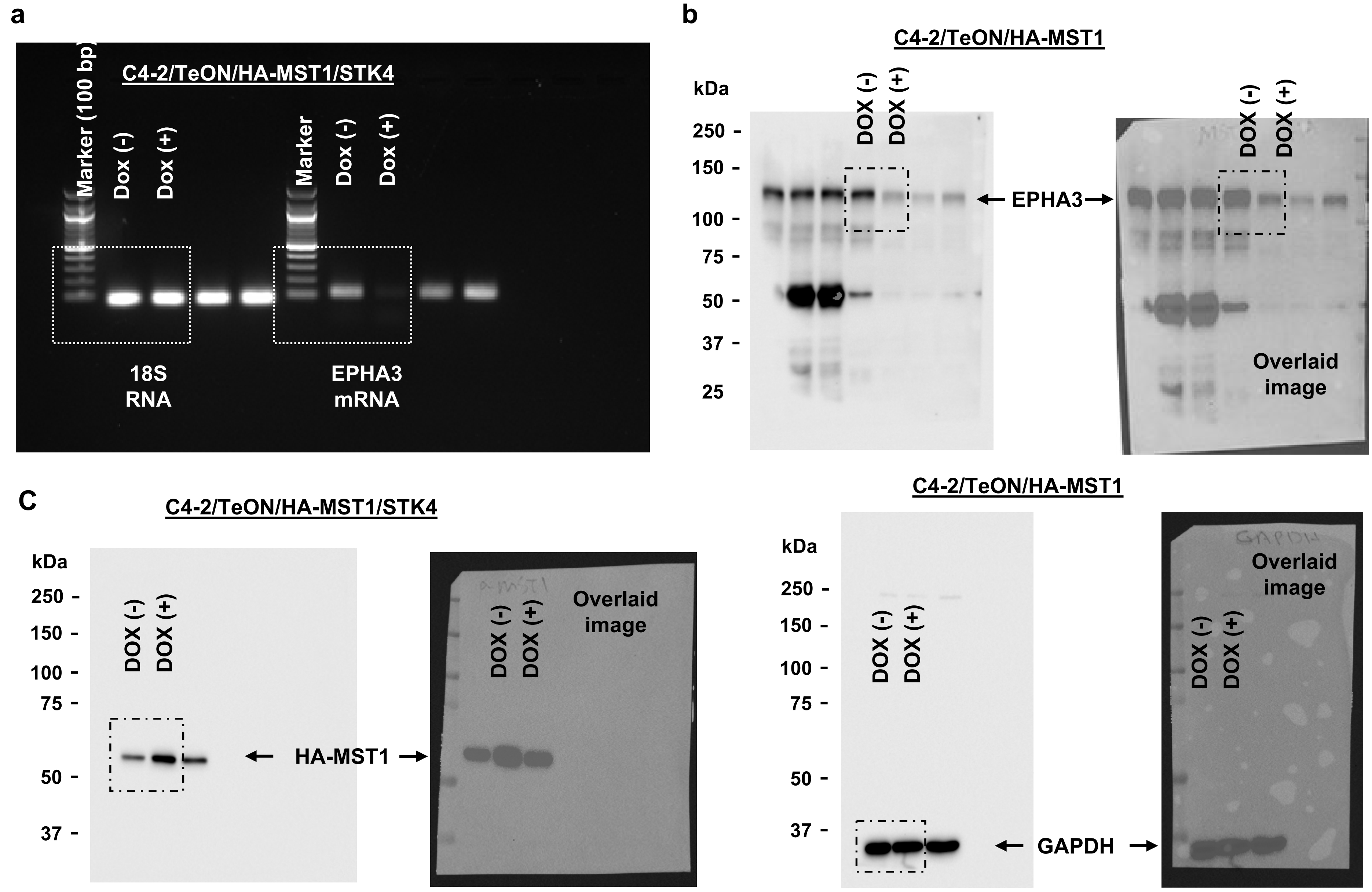

Figure S4. Images of full-length gel and blots. The gel with a dotted box in in Figure S2a were cropped to generate figure 2c and the blots in Figure S4b and S4c were cropped to generate Figure 2c in the main manuscript. Note that it is difficult to capture the edges of the membrane because images were not captured using X-ray films. Micrographs provided were digitally captured. The overlaid image was different exposure that shows the edges of the membrane with the corresponding blot.

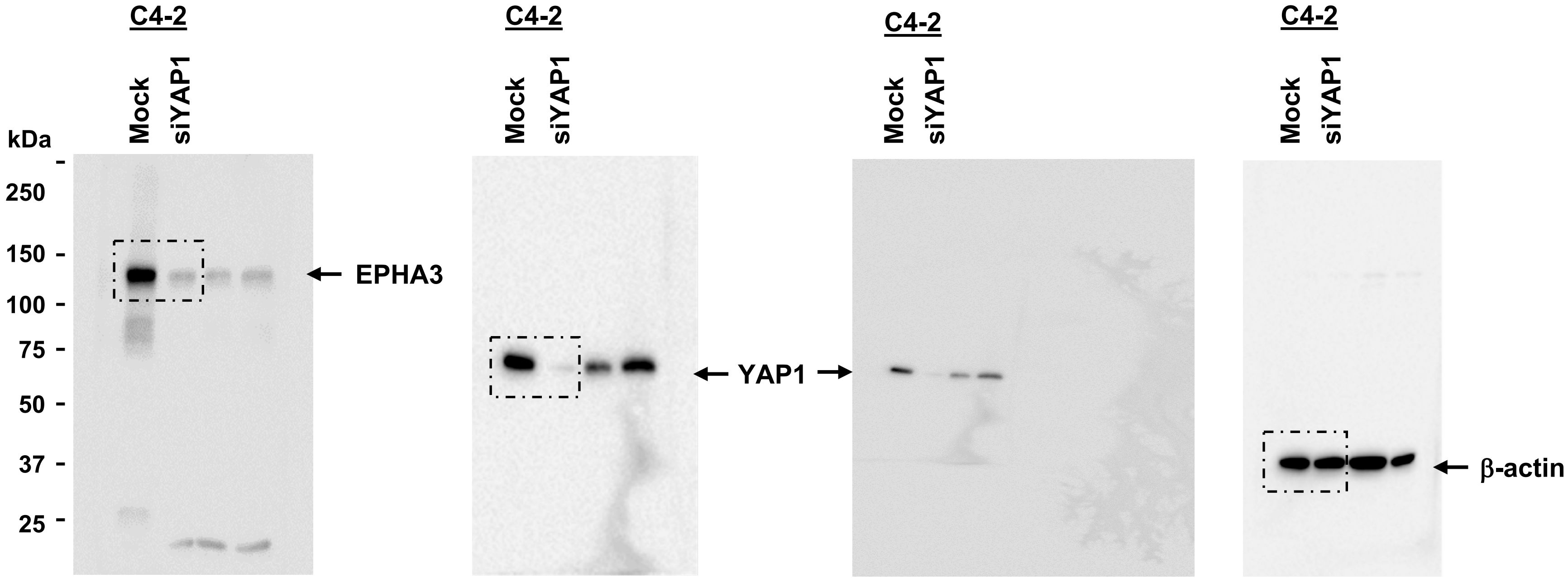

Figure S5. Full-length blots. The blots with a dotted box in were cropped to generate figure 2d in the main manuscript. Note that it is difficult to capture the boundaries of the membrane because images were not captured using X-ray films. Micrographs provided were digitally captured.

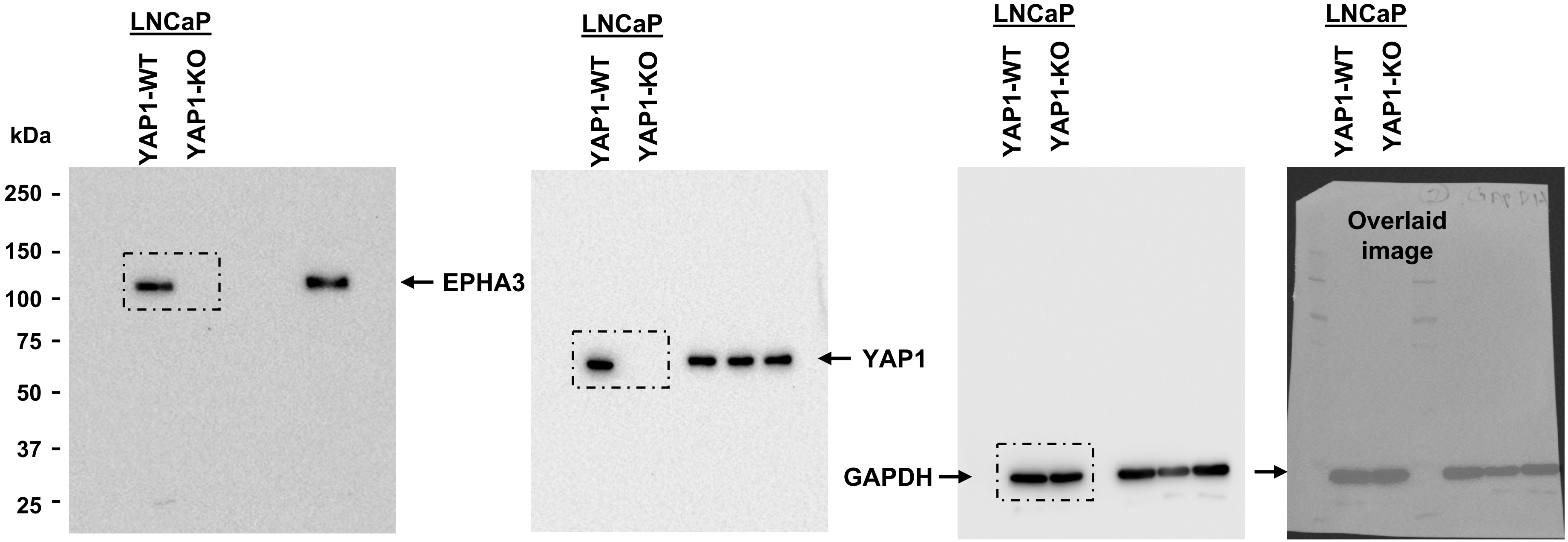

Figure S6. Images of full-length blots. The blots with a dotted box were cropped to generate figure 2e in the main manuscript. The overlaid image was different exposure that shows the boundaries of the membrane with the corresponding blot. Note that it is difficult to capture the edges of the membrane because images were not captured using X-ray films. Micrographs provided were digitally captured.

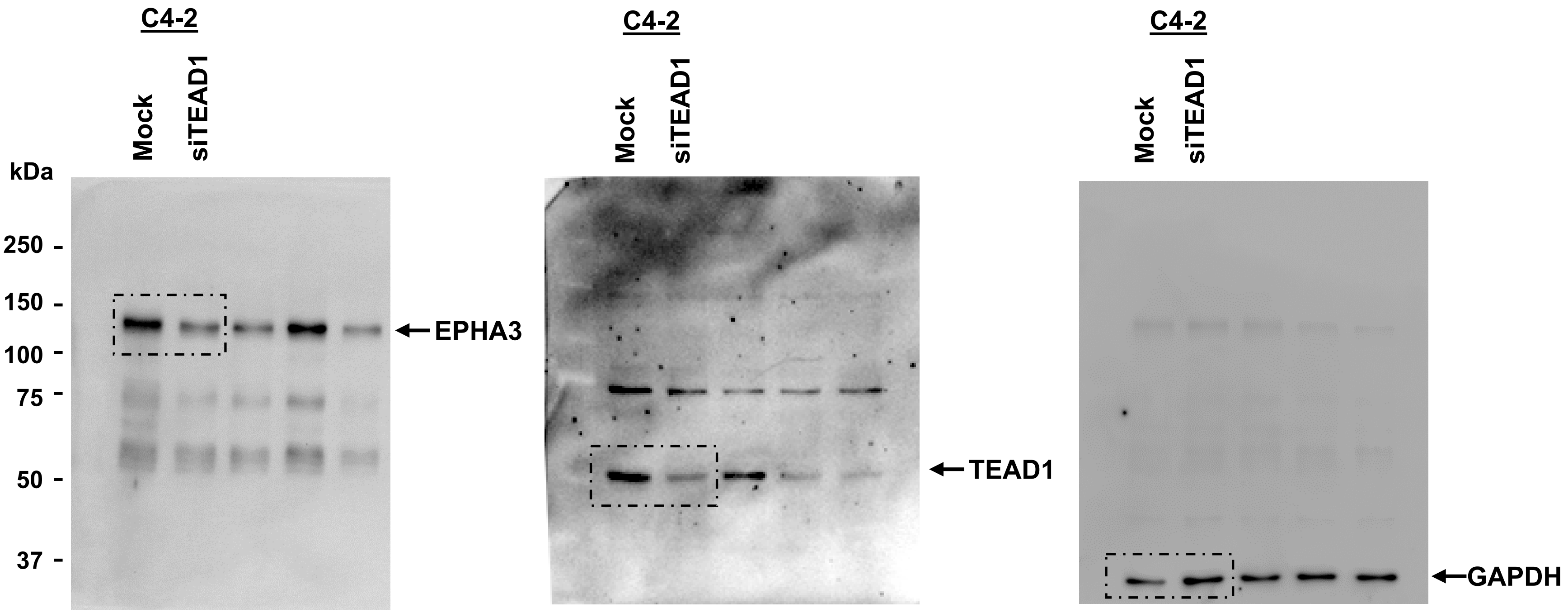

Figure S7. Images of full-length blots. The blots with a dotted box were cropped to generate figure 2f in the main manuscript. Note that it is difficult to capture the boundaries of the membrane because images were not captured using X-ray films. Micrographs provided were digitally captured.

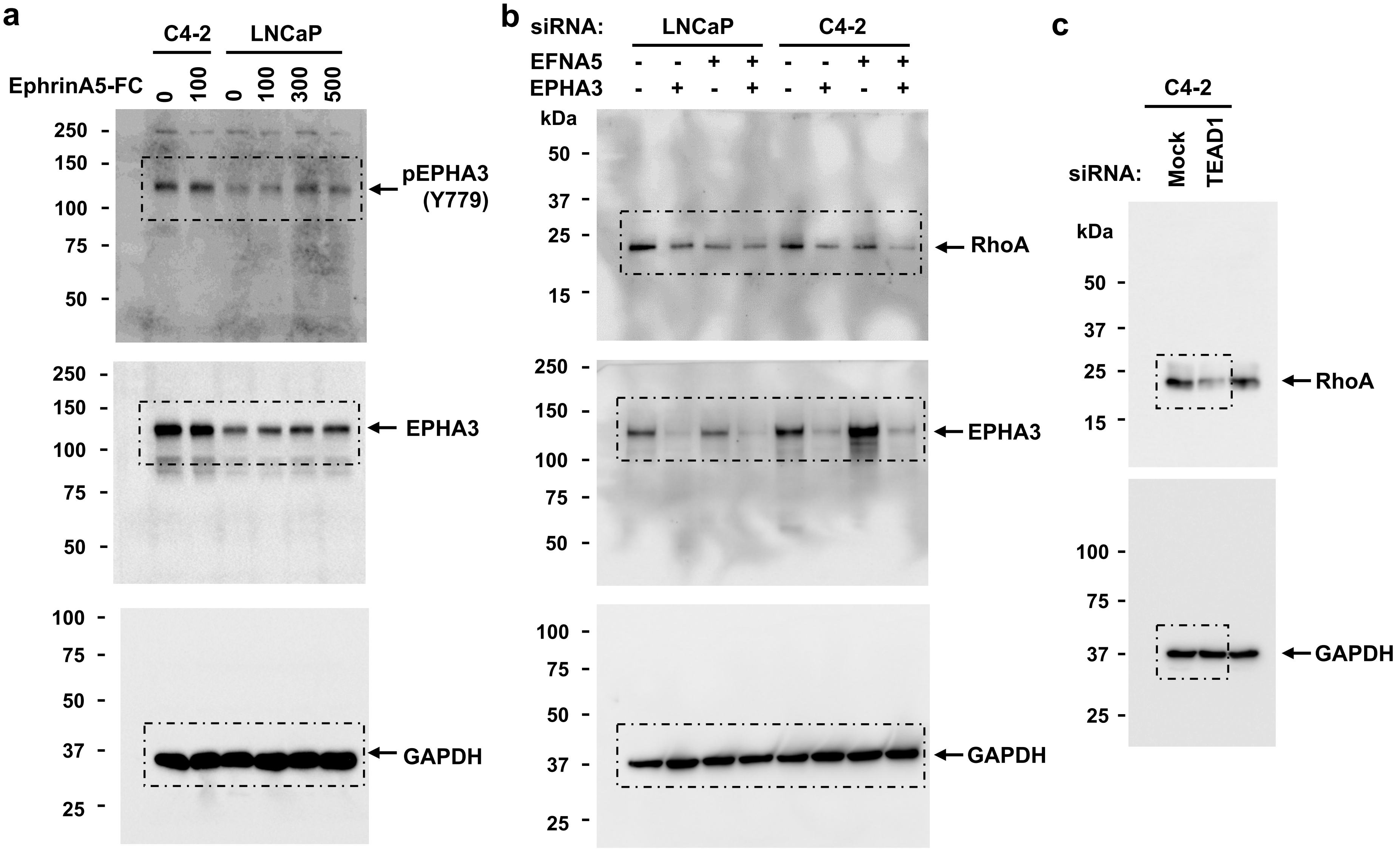

Figure S8. Images of full-length blots. The blots with a dotted box were cropped to generate figures 5a, 5c, and 5e in the main manuscript. Note that the RhoA protein was analyzed by 12% SDS-PAGE gel, and other proteins were analyzed by 8% SDS-PAGE gel. Note that it is difficult to capture the boundaries of the membrane because images were not captured using X-ray films. Micrographs provided were digitally captured.

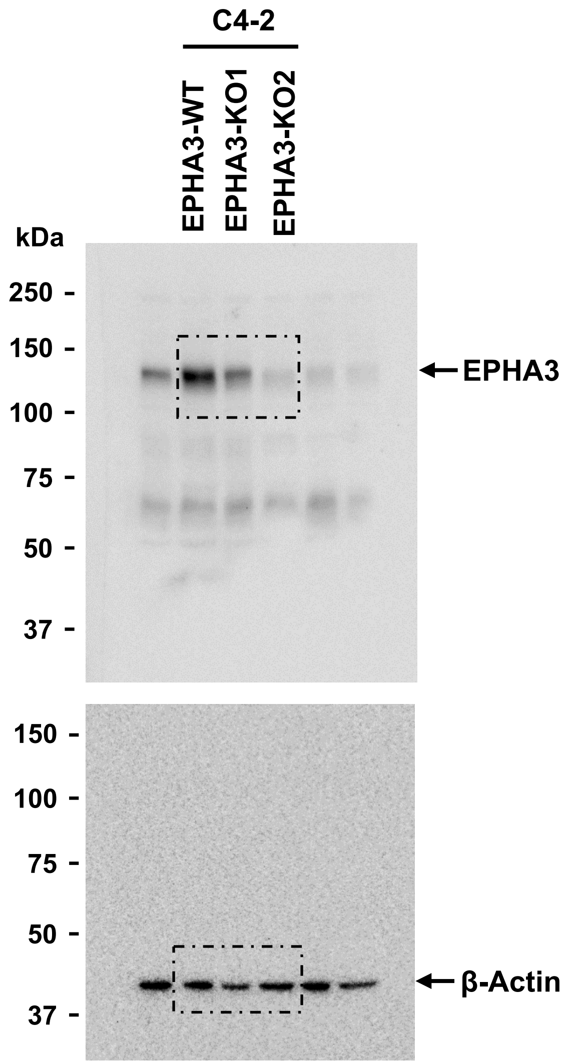

Figure S9. Images of full-length blots. The blots with a dotted box were cropped to generate figure 6a in the main manuscript. Note that it is difficult to capture the boundaries of the membrane because images were not captured using X-ray films. Micrographs provided were digitally captured. The beta-actin blot was acquired a quick with view image mode, which resulted in a contrast image.

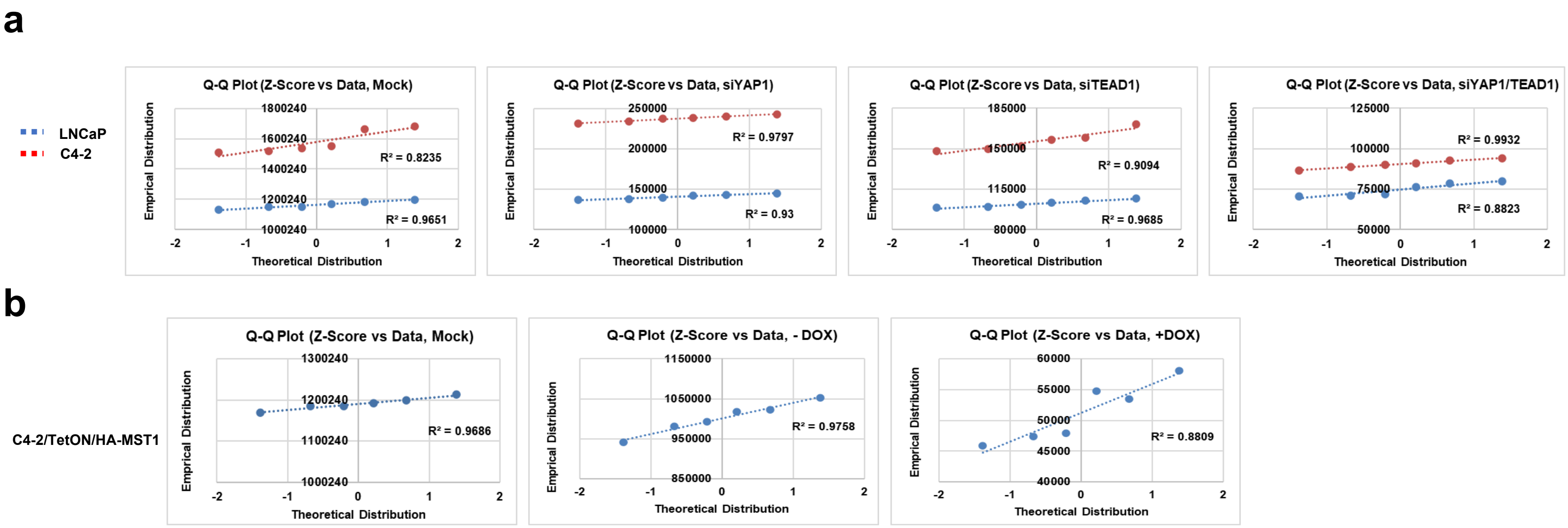

Figure S10. Q-Q plot (scatter plot) of the luciferase reporter data sets in LNCaP and C4-2 cells (a) and C4-2/TeON-HA-MST1 cells (b). The graphs show the probability of normal distribution of the data (y-axis) as a function of z-score (x-axis). The statistical analysis was performed using Microsoft excel.

Cell-cell interaction data (a)

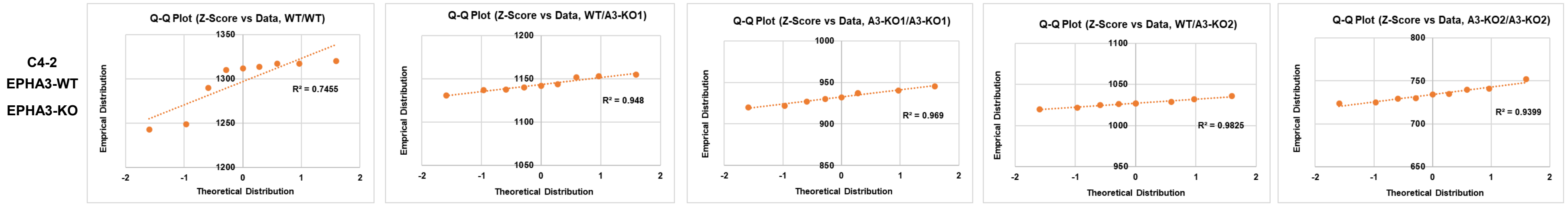

Wound healing data (b)

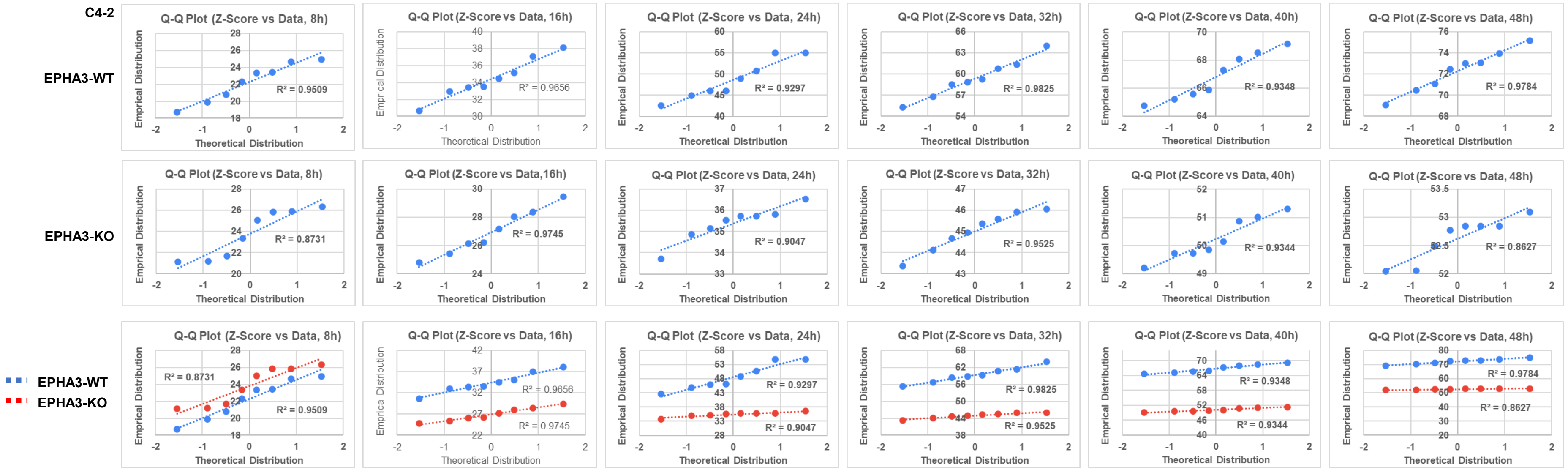

Figure S11. Q-Q plot (scatter plot) of the cell-cell interaction (a) and wound healing (a) data sets. The graphs show the probability of normal distribution of the data (y-axis) as a function of z-score (x-axis). The statistical analysis was performed using Microsoft excel.
