# Supplementary material for: The Hippo effector YAP1/TEAD1 regulates EPHA3 expression to control cell contact and motility
Source: Sci Rep. 2022 Mar 9;12:3840. doi: 10.1038/s41598-022-07790-4 (PMC8907295; doi:10.1038/s41598-022-07790-4)
Supplement: Supplementary file 2 — Supplementary Information 2. [file 41598_2022_7790_MOESM2_ESM.pdf]

Identification of TEAD binding sites or TEAD responsive elements (TREs) within human EPHA3 promoter DNA sequence.

1. In the https://epd.epfl.ch/ web portal, we search for the human (*H. sapiens*) EPHA3 gene.

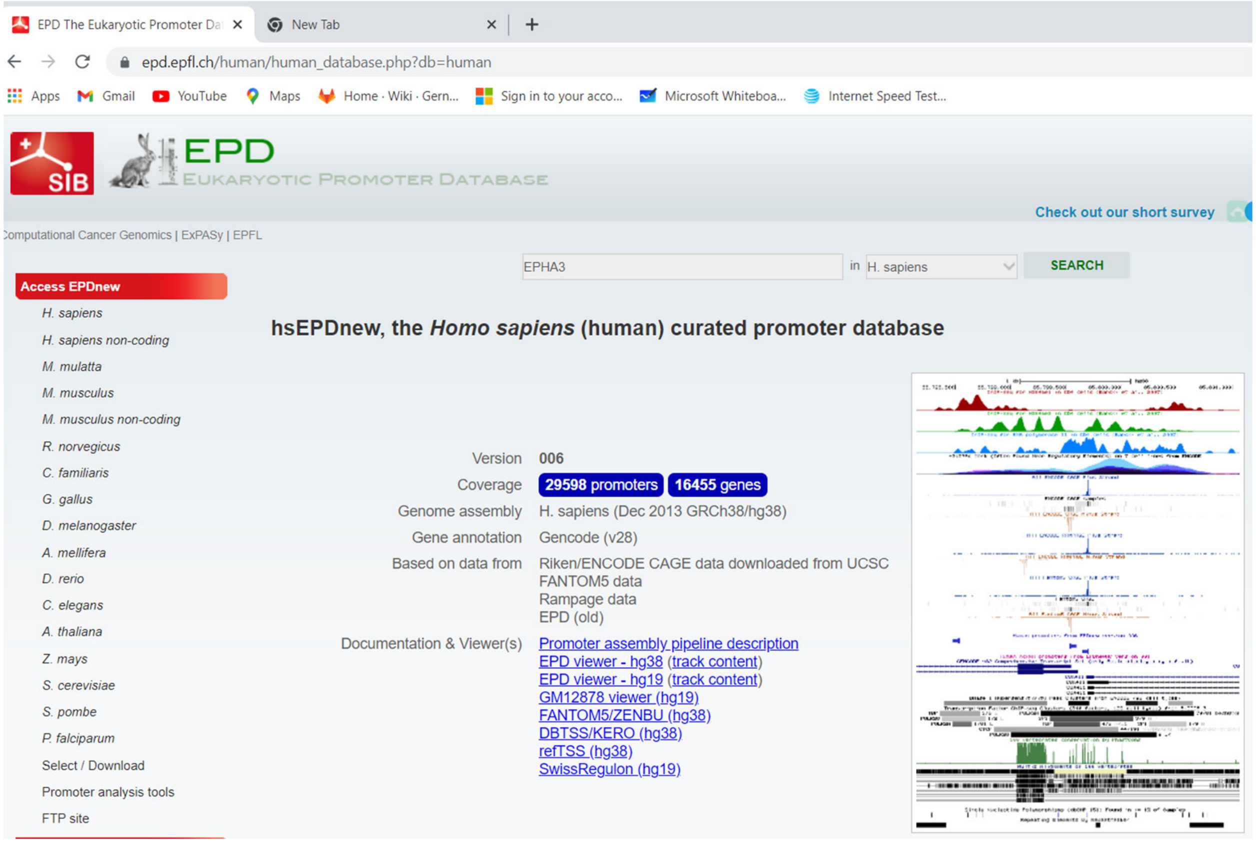

The screenshot below shows the graphical presentation of a portion of the search results as proof.

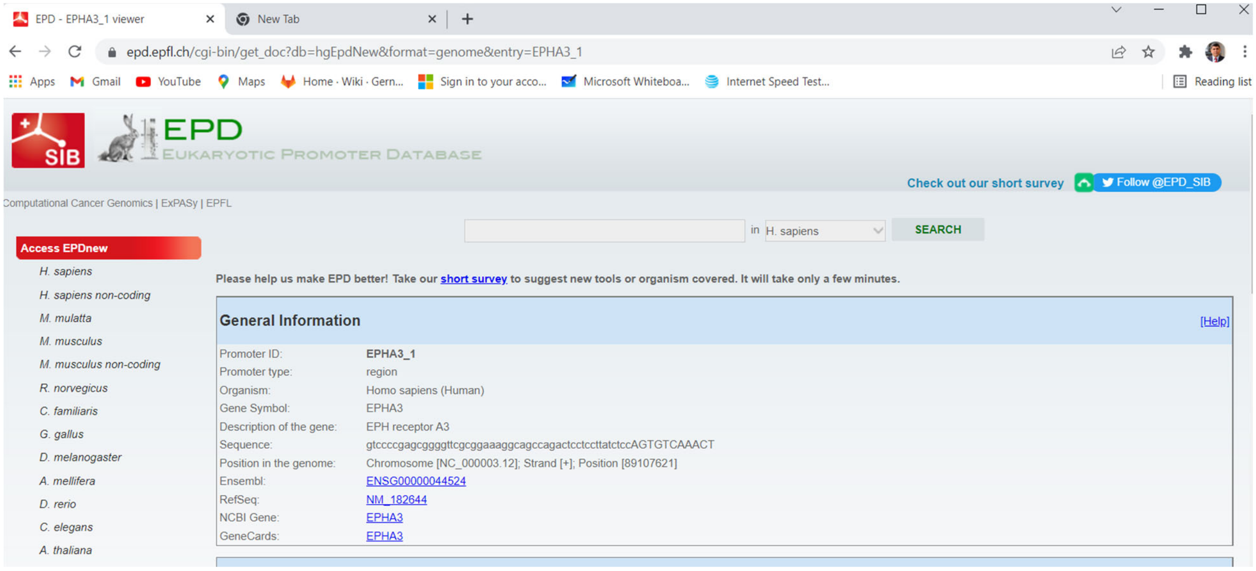

Predicted TSS: gtccccgagcggggttcgcggaaaggcagccagactcctccttatctccAGTGTCAAACT

2. In the Search Motive Tool on the same window, we set the Library to Transcription Factor Motifs (powered by JASPER) and Motif to TEAD1. The search the TEAD1 binding sites with the EPAH3 promoter from -5000 to 100 bp relative to Transcriptional Start Site (TSS) and cut-off p-value of 0.0001 (1st) and 0.00001 (2nd for more stringent statistics). We repeated the research for TEAD2, TEAD3, and TEAD4 using the same search criteria. The screenshots below show the graphical presentation of the predicted TEAD1 binding site.

The search resulted in three predicted TEAD binding sites located at the -3551, -1841, and -887 DNA regions relative to TSS when we set the cut-off p-value to 0.00001.

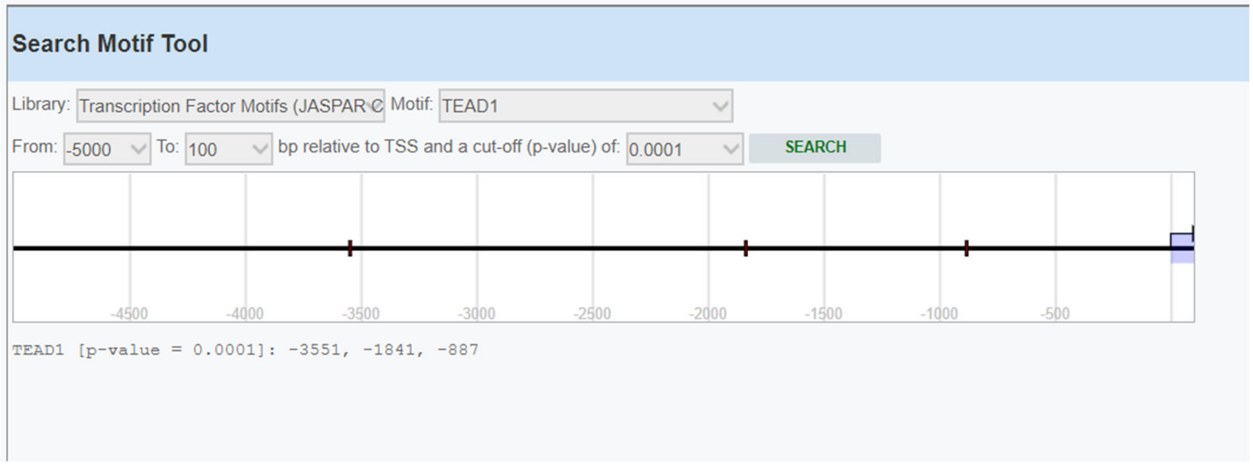

The search resulted in a predicted TEAD1 binding site located at the -3551 DNA region relative to TSS when the cut-off p-value was set to 0.00001.

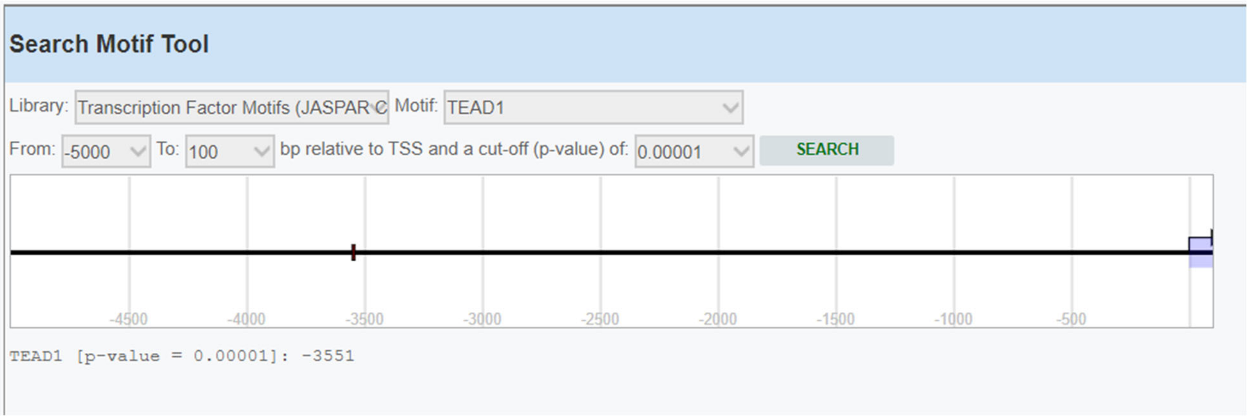

3. Using Sequence Retrieval Tool (on the same window), we obtained the DNA sequence of EPHA3 promoter from -5000 to 100 bp relative to TSS.

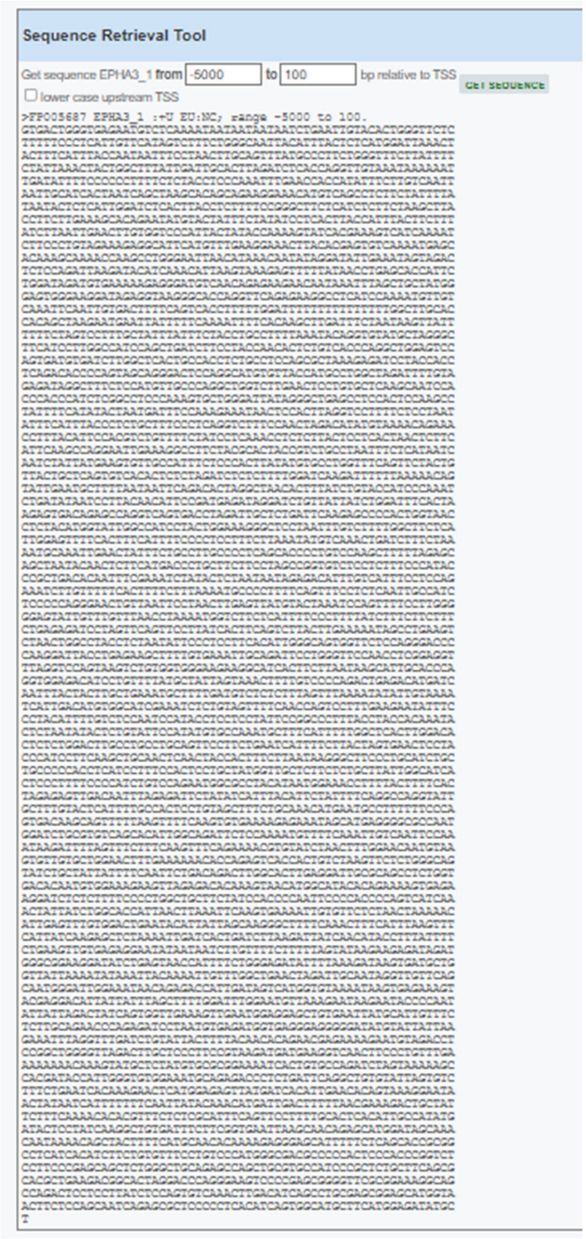

>FP005687 EPHA3_1 :+U EU:NC; range -5000 to 100.
GTGACTGGGTGAGAATGTCTCAAAATAATAATAATAATCTGAATTGTACACTGGGTTCTC
TTTTTCCCTCATTGTTCATAGTCTTTCTGGGCAATTACATTTACTCTCATGGATTAAACT
ACTTTCATTTACCAATAATTTCCTAACTTGCAGTTTATGCCCTTCTGGGTTTCTTATTTT
CTATTAAACTACTGGCTTTATTGATTGCACTTAGATCTCACCAGGTTGTAAATAAAAAAT
TGATATTTTCCCCCCTTTTCTCTACCTCCCAAATTTGAACCACCATATTTCTTGTCAATT
AATTGCATCACTAATCAGCTAAGCACAGCAGAAGGAAACATGTCAGCCTCTTCTATTTTA
TAATACTCTCATTGGATCTCACTTACCTCTTTTCCGGGCTTCTCATCTCTTCTAAGCTTA
CCTTCTTGAAAGCACAGAATATGTACTATTTCTATATCCTCACTTACCATTTACTTCTTT
ATCTTAATTGAACTTGTGGTCCCATTACTATACCAAAAGTATCACGAAAGTCATCAAAAT
CTTCCCTGTAGAAAGAGGCATTCATGTTTGAAGGAAACTTACACGAGTGTCAAAATGAGC
ACAAAGCAAAACCAAGCCTGGGAATTAACATAAACAATATAGGATATTGAAATAGTAGAC
TCTCCAGATTAAGATACATCAAACATTAAGTAAAGAGTTTTTATAACCTGAGCACCATTC
TGGATAGATGTGAAAAAGAGGGATGTCAACAGAGAAGAACAATAAATTTAGCTGCTATGG

GAGTGGGAAGGATAGAGGTAAGGGCACCAGGTTCAGAGAAGGCCTCATCCAAAATGTTGT
CAAATTCAATTGTGACTTTTCAGTCACCTTTTTGGATTTTTTTTTTTTTTTTGGCTTGCAC
CACAGCTAAGAATGAATTATTTTTCAAAATTTTCACAAGCTTGATTTCTAATAAGTTATT
TTTTCTAGTCCTTTGCTATTTATTTCTACCTGCCTTTTAAATACAGGTGTATGCTAGGGC
TTCATCCTTGGCCATCCAGCTGATCTTCCTACCAACACTCTGTCACCCAGGCTGGAGTCC
AGTGATGTGATCTTGGCTCACTGCCACCTCTGCCTCCAGCGCTAAAGAGATCCTACCACC
TCAGACACCCCAGTAGCAGGGACTCCAGGCATGTGTTACCATGCCTGGCTAGATTTTGTA
GAGATAGGCTTTCTCCATGTTGCCCAGGCTGGTCTTGAACTCCTGTGCTCAAGCAATCCA
CCCACCCATCTCGGCCTCCCAAAGTGCTGGGATTATAGGGCTGAGCCTCCACTCCAAGCC
TATTTTCATATACTAATGATTTCCAAAGAAATAACTCCACTTAGGTCCTTTTCTCCTAAT
ATTTCATTTACCCTCTGCTTTCCCTCAGGTCTTTCCAACTAGACATATGTAAAACAGAAA
CCTTTACATTCCACGTCTGTTTTCTATCCTCAAACCTCTCTTACTCCTCACTAACTCTTC -3551 DNA seq underlined
ATTCAAGCCAGGAATTGAAAGGCCTTCTACGCACTACCGTCTGCCTAATTTCTCATAATC
AATCTATTATGAAGTGTTGCCATTTCTCCCACTTATATGTGCCTGGTTTCAGTTCTACTG
TTACTGCTCAGTGTCACACTCTCTAGATCTCTCTTTTGGATCAAGATTTTTTAAAAACAG
TATTGAATGCTTTTAATAATTCAGACACTAGGCTAACACTTTATCTGTACCATCCCAAAT
CTGATATAATCCTTACAACATTCCGATGAGATAGGATCTGTTATTATCTGGATTTCACTA
AGAGTGACAGAGCCAGGTCAGTGACCTAGATTGCTCTGATTCAAGAGCCCCACTGGTAAC
CTCTACATGGTATTGGCCATCCTACTGGAAAGGGCTCCTAATTTGTCTTTTGGCTTCTCA
TTGGAGTTTTCACTTTCATTTTCCCCTCCTTTCTTAAATATGTCAAACTGATCTTTCTAA
AATGCAAATTGAACTATTTCTGCCTTGCCCCTCAGCACCCCTGTCCAAGCTTTTTAGAGC
AGCTAATACAACTCTTCATGACCCTGCTTCTTCCTAGCCGGTGTCTCCTCTTTCCCATAC
CCGCTGACACAATTTCGAAATCTATACTCTAATAATAGAGACATTTGTCATTTCCTCCAG
AAATCTTGTTTTTCACTTTTCTTTAAAATGCCCCTTTTCAGTTTCCTCTCAATTGCCATC
TCCCCCAGGGAACTGTTAATTCCTAACTTGAGTTATGTACTAAATCCAGTTTTCCTTGGG
GGAGTATTGTTTGTTTAACCTAAAATGGTCTTCTCATTTTCCCTTTTATCTTTCTTCTTT
CTGAGAGATCCTAGTTCAGTTCCTTATCACTTCAGTCTTACTTGAAAAATAGCCTGAAGT
CTAACTGGCCTACCTCTAATATTCCCTCCTTCACATTGGGCAGTGGTTCTCCAGGGACCC
CAAGGATTACCTGAGAAGCTTTTGTGAAATTGCAGATTCCTGGGTTCCAACCTCGGAGGT -1841 DNA seq underlined
TTAGGTCCAGTAAGTCTGTGGTGGGAAGAAGGCATCACTTCTTAATAAGCATTGCACCCA
GGTGGAGACATCCTGTTTTATGCTATTAGTAAACTTTTGTCCCCAGACTGAGACATGATC
AATTTACTACTTGCTGAAATGCTTTTGATGTCTCTCTTTAGTTTAAAATATATTGTAAAA
TCATTGACATGTGGCATCGAAATCTCTGTAGTTTTCAACCAGTCCTTTGAAGAATATTTC
CCTACATTTTGTCTCCAATCCATACCTCCTCCTATTCCGGCCCTTTACCTACCACAAATA
CTCTAATATACTCTGTATTCCATATGTGCCAAATGCTTTCATTTTTGGCTCACTTGGACA
CTCTCTGGACTTGCCTGCCTGCAGTTCCTTCTGAATCATTTTCTTACTAGTGAACTCCTA
CCCATCCTTCAAGCTGCAACTCAACTACCACTTTCTTAATAAGGGCTTCCCTGCATCTGC
TGCCCCCACCTCATCCTTTCCACTCCTGCTATGGTTGCTCTTCTCTGCTTATTGGCATCA
CTCCCTTTTCCCCATCTGTCCAGAATGGCGCCTACATAATGGAAACCTTTTACTTTTCAC
TAGAGAGTTGACAATTTAGAGATTCTATATCATTTACATTCTATTTTCAGGCCAGGTATT
GCTTTGTACTCATTTTGCCACTCCTGTAGCTTTCTGCAAACATGAATGCCTTTTTTCCCA
GTGACAAGCAGTTTTTAAGTTTTCAAGTGTGAAAAGAGAAATAGCATGAGGGGCGCCAAT
GGATCTGCGTGTCAGCACATTGGCAGATTCTCCAAAATGTTTTCAAATTGTCAATTCCAA
ATAAGATTTTAGTTTCTTTCAAGTTTCAGAAAACGTGTATCTAACTTTGGAACAATGTAA
GTGTTGTGCTGGAACTTTGAAAAAACACCAGAGTCACCACTGTCTAAGTTCTCTGGGCAG
TATCTGCTATTATTTTCAATTCTGACAGACTTGGCACTTGAGGATTGCGCAGCCTCTGGT
GACACAATGTGGAAAGAAGTTAGAGACACAAAGTAACATGGCATACACAGAAAAGTGAGA
AGGATCTCTCTTTTCCCCTGGCTGCTTCTATCCACCCCAATTCCCCACCCCAGTCATCAA
ACTATTATCTGGCACCATTAACTTAAATTCAAGTGAAAATTGTGTTCTCTAACTAAAAAC
ATTGAGTTTGTGGACTGAATACATTATTAGCAAGGGCTTTTCAAACTTTCATTTAAGTTT
CATTATCAAGAGCTCTAAAATTGATCACTGATCTTAAGATTATCAACATACCTTTATTTT
CTGAAGTTGTGAGAGGAATATAATAATCTTGTTTTCTTTTTAGTATAAGAAGAGATAGAT
GGGCGGAAGGATATCTGAGTAACCATTTTCTGGGAGATATTTTAAAGATAAGTGATGCTG
GTTATTAAAATATAAATTACAAAATTGTTTGGCTGAACTAGATTGCAATAGGTTGTTCAG
CAATGGGATTGGAAATAACAGAGACCATTGATAGTCATGGTGTAAAATAAGTGAGAAAGT
ACGAGGACATTATTATTTAGCTTTTGGATTTGGAATGTTAAAGAATAAGAATACCCCAAT -887 DNA seq underlined
ATTATTAGACTATCAGTGGTTGAAAGTTGAATGGAGGAGCTGTGAATTATGCATTGTTTC
TCTTGCAGAACCCAGAGATCCTAATGTGAGATGGTGAGGGAGGGGGATATGTATTATTAA

```
GAAATTTAGGTTTGATCTGTATTACTTTTACAACACAGAACGAGAAAAGAATGTAGACCT
CCGGCTGGGGTTAGACTTGCTCCCTTCCGTAAGATGATGAAGGTCAACTTCCCTGTTTGA
AAAAAAACAAAGTATGCTCTATGTGCGCGGAAAATCACTGTGCCAGATCTAGTAAAAAGC
CACGATACCATTGGGTGTGGAAATGCAGAGACCCTCTGATTCAGGCTGTGTATTAGTGTC
TTTCTGAATCACAAAGAACTCATGGAGAGTTATGATCACATTGAACACAGTAAAGGAATA
ACTATAATCATTTTTTTCAATTATACAAACATGATTGACTTTTTAACGAAAGACTGCTAT
TCTTTCAAAACACACGTTTCTCTCGCATTTCAGTTCCTTTTGCACTCACATTGCCATATG
ATACTCCTATCAAGGCTGTGATTTCTTCGGTGAATTAAGCAACAGAGCATGGATAGCAAA
CAATAAAACAGCTACTTTTCATGCAACACAAAAGAGGGAGCATTTTTCTCAGCACCGCGG
CCTCATCACATCTTCTGTGTTTCCTGTCCCATGGGCGACGCCCCCACTCCCACCCGGTCT
CCTTCCCGAGCAGCTCTGGGCTGCAGAGCCAGCTGCGTGCCATCCCGCTCTGCTTCAGCG
CACGCTGAAGACGGCACTAGGACCCAGGGAAGTCCCCGAGCGGGGTTCGCGGAAAGGCAG Promoter Region
CCAGACTCCTCCTTATCTCCAGTGTCAAACTTGACATCAGCCTGCGAGCGGAGCATGGTA
ACTTCTCCAGCAATCAGAGCGCTCCCCCTCACATCAGTGGCATGCTTCATGGAGATATGC
T
```

4. Then, we manually locate the predicted TEAD1 binding sites -3551, -1841, and -887 relative to TSS.
5. We copied and past about 90 bp DNA sequence flanking of the left and right of the TEAD binding site into the https://jaspar.genereg.net/ search tool (Scan box) to verify the presence of TEAD1 binding site at the predicted DNA region using "relative score threshold" 80%. Below is the screenshot of the JASPAR analysis results for the TEAD1 core binding sites and their logo with matrix ID.

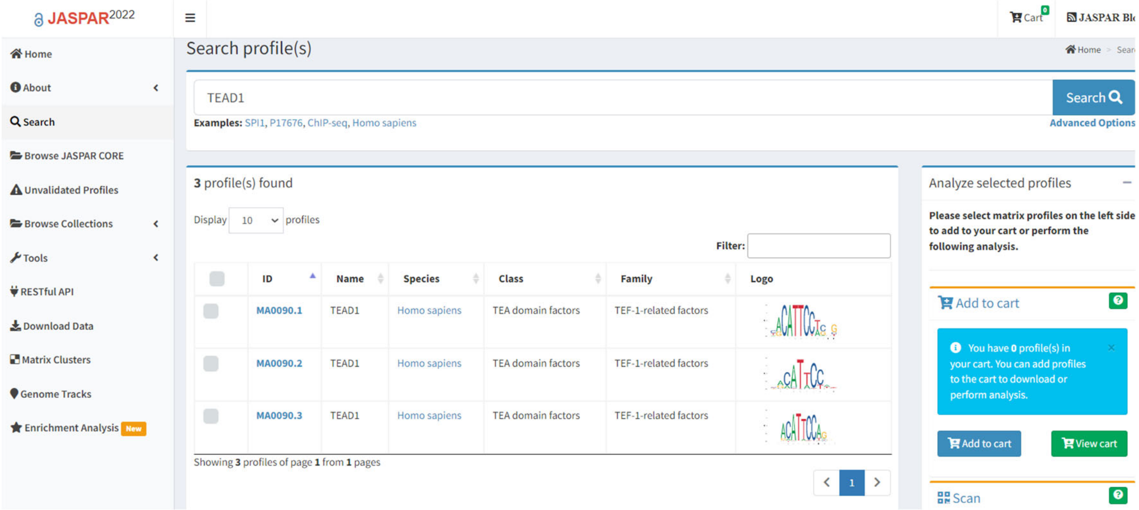

Matrix ID: MA0090.1

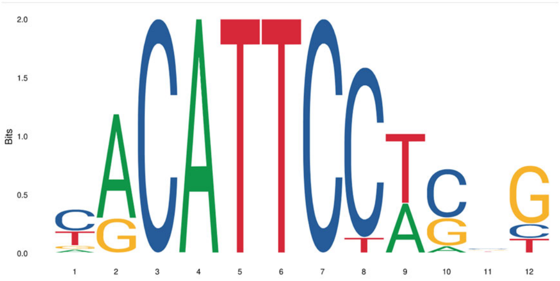

Matrix ID: MA0090.2

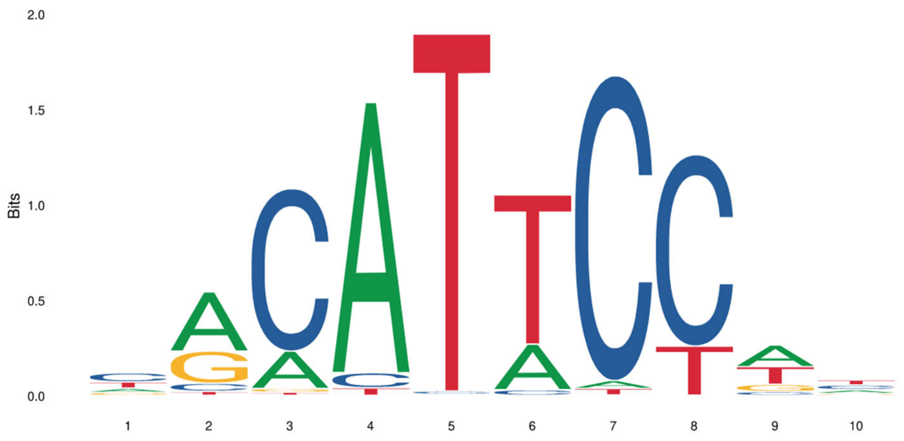

Matrix ID: MA0090.3

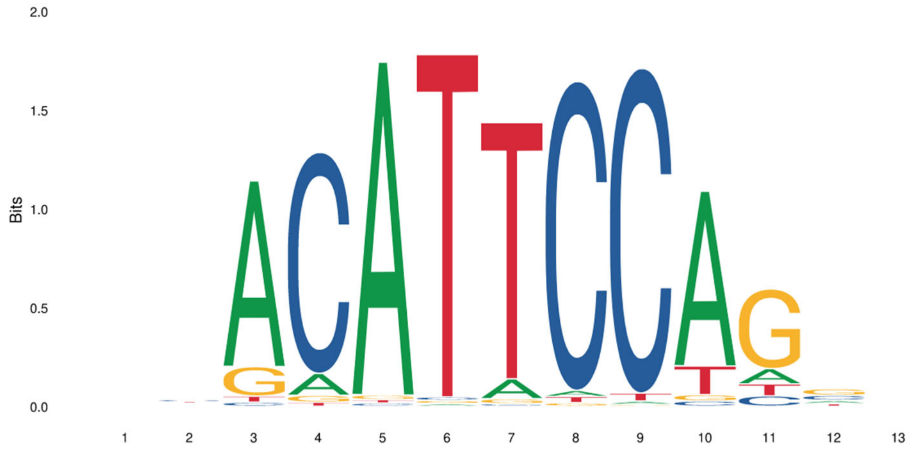

>Sequence -3551 (5' to 3' positive strand)
ATTTCATTTACCCTCTGCTTTCCCTCAGGTCTTTCCAACTAGACATATGTAAAACAGAAACCTTTACATTCCACGTCT
GTTTTCTATCCTCAAACCTCTCTTACTCCTCACTAACTCTTCATTCAAGCCAGGAATTGAAAGGCCTTCTACGCACTA
CCGTCTGCCTAATTTCTCATAATC

Below is the screenshot of the JASPAR analysis results for the predicted TEAD1 binding site (with the high score as indicated arrow in red) at the -3551 DNA region of the EPAH3 promoter.

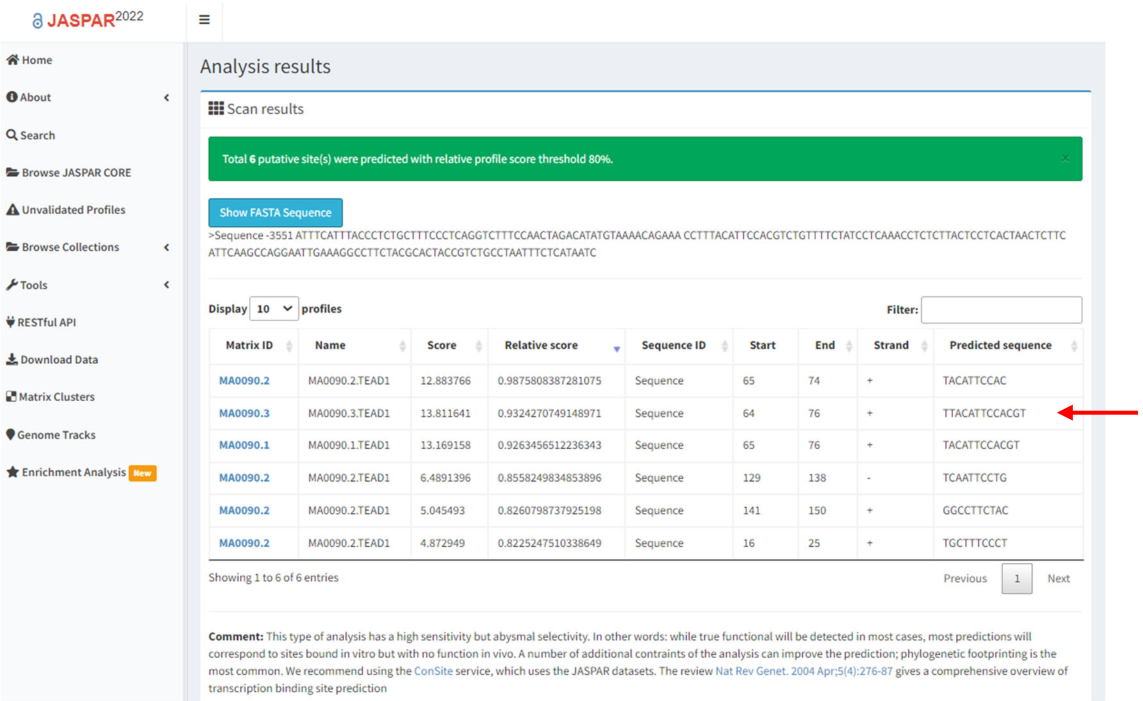

JASPAR2022

Home
About
Search
Browse JASPAR CORE
Unvalidated Profiles
Browse Collections
Tools
RESTful API
Download Data
Matrix Clusters
Genome Tracks
Enrichment Analysis New

Analysis results

Scan results

Total **6** putative site(s) were predicted with relative profile score threshold 80%.

Show FASTA Sequence

>Sequence -3551 ATTTCATTTACCCTCTGCTTTCCCTCAGGTCTTTCCAACTAGACATATGTAAAACAGAAA CCTTTACATTCCACGTCTGTTTTCTATCCTCAAACCTCTCTTACTCCTCACTAACTCTTC ATTCAAGCCAGGAATTGAAAGGCCTTCTACGCACTACCGTCTGCCTAATTTCTCATAATC

Display 10 profiles Filter:

Matrix ID	Name	Score	Relative score	Sequence ID	Start	End	Strand	Predicted sequence
MA0090.2	MA0090.2.TEAD1	12.883766	0.9875808387281075	Sequence	65	74	+	TACATTCCAC
MA0090.3	MA0090.3.TEAD1	13.811641	0.9324270749148971	Sequence	64	76	+	TTACATTCCACGT
MA0090.1	MA0090.1.TEAD1	13.169158	0.9263456512236343	Sequence	65	76	+	TACATTCCACGT
MA0090.2	MA0090.2.TEAD1	6.4891396	0.8558249834853896	Sequence	129	138	-	TCAATTCCTG
MA0090.2	MA0090.2.TEAD1	5.045493	0.8260798737925198	Sequence	141	150	+	GGCCTTCTAC
MA0090.2	MA0090.2.TEAD1	4.872949	0.8225247510338649	Sequence	16	25	+	TGCTTTCCCT

Showing 1 to 6 of 6 entries Previous 1 Next

Comment: This type of analysis has a high sensitivity but abysmal selectivity. In other words: while true functional will be detected in most cases, most predictions will correspond to sites bound in vitro but with no function in vivo. A number of additional contraints of the analysis can improve the prediction; phylogenetic footprinting is the most common. We recommend using the ConSite service, which uses the JASPAR datasets. The review Nat Rev Genet. 2004 Apr;5(4):276-87 gives a comprehensive overview of transcription binding site prediction

>Sequence -1841 (5' to 3' positive strand)
CTAACTGGCCTACCTCTAATATTCCCTCCTTCACATTGGGCAGTGGTTCTCCAGGGACCCCAAGGATTACCTGAGAA
GCTTTTGTGAAATTGCAGATTCCTGGGTTCCAACCTCGGAGGTTTAGGTCCAGTAAGTCTGTGGTGGGAAGAAGG
CATCACTTCTTAATAAGCATTGCACCCA

Below is the screenshot of the JASPAR analysis results for the predicted TEAD1 binding site (with the high score as indicated arrow in red) at the -1841 DNA region of the EPAH3 promoter.

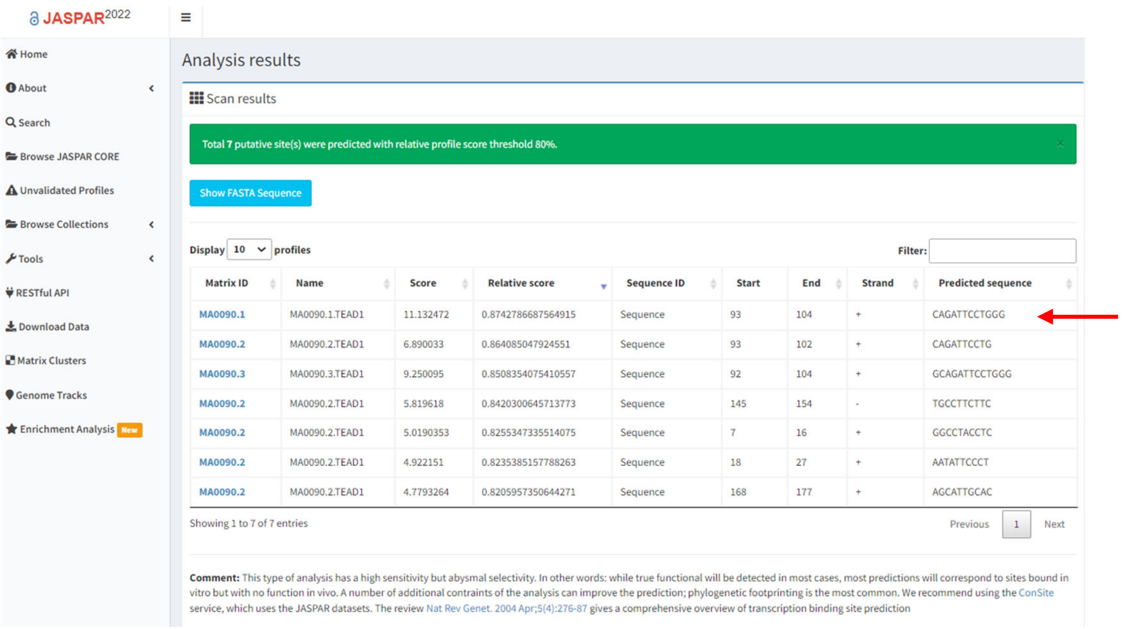

JASPAR2022

Home
About
Search
Browse JASPAR CORE
Unvalidated Profiles
Browse Collections
Tools
RESTful API
Download Data
Matrix Clusters
Genome Tracks
Enrichment Analysis New

Analysis results

Scan results

Total **7** putative site(s) were predicted with relative profile score threshold 80%.

Show FASTA Sequence

Display 10 profiles Filter:

Matrix ID	Name	Score	Relative score	Sequence ID	Start	End	Strand	Predicted sequence
MA0090.1	MA0090.1.TEAD1	11.132472	0.8742786687564915	Sequence	93	104	+	CAGATTCCTGGG
MA0090.2	MA0090.2.TEAD1	6.890033	0.864085047924551	Sequence	93	102	+	CAGATTCCTG
MA0090.3	MA0090.3.TEAD1	9.250095	0.8508354075410557	Sequence	92	104	+	GCAGATTCCTGGG
MA0090.2	MA0090.2.TEAD1	5.819618	0.8420300645713773	Sequence	145	154	-	TGCCTTCTTC
MA0090.2	MA0090.2.TEAD1	5.0190353	0.8255347335514075	Sequence	7	16	+	GGCCTACCTC
MA0090.2	MA0090.2.TEAD1	4.922151	0.8235385157788263	Sequence	18	27	+	AATATTCCCT
MA0090.2	MA0090.2.TEAD1	4.7793264	0.8205957350644271	Sequence	168	177	+	AGCATTGCAC

Showing 1 to 7 of 7 entries Previous 1 Next

Comment: This type of analysis has a high sensitivity but abysmal selectivity. In other words: while true functional will be detected in most cases, most predictions will correspond to sites bound in vitro but with no function in vivo. A number of additional contraints of the analysis can improve the prediction; phylogenetic footprinting is the most common. We recommend using the ConSite service, which uses the JASPAR datasets. The review Nat Rev Genet. 2004 Apr;5(4):276-87 gives a comprehensive overview of transcription binding site prediction

>Sequence -887 (5’ to 3’ positive strand)
CAATGGGATTGGAAATAACAGAGACCATTGATAGTCATGGTGTAAAATAAGTGAGAAAGTACGAGGAC
ATTATTATTTAGCTTTTGGATTTGGAATGTTAAAGAATAAGAATACCCCAATATTATTAGACTATCAGTGG
TTGAAAGTTGAATGGAGGAGCTGTGAATTATGCATTGTTTC

Below is the screenshot of the JASPAR analysis results for the predicted TEAD1 binding site (with the high score as indicated arrow in red) at the - 887 DNA region of the EPAH3 promoter.

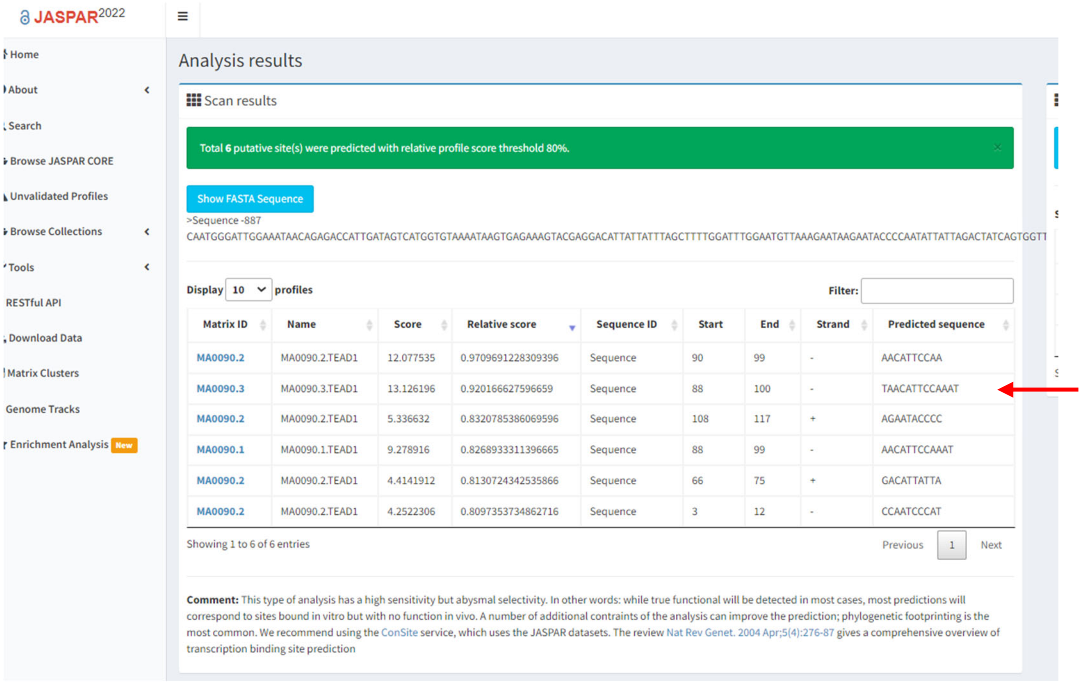

JASPAR2022

Home
About
Search
Browse JASPAR CORE
Unvalidated Profiles
Browse Collections
Tools
RESTful API
Download Data
Matrix Clusters
Genome Tracks
Enrichment Analysis New

Analysis results

Scan results

Total 6 putative site(s) were predicted with relative profile score threshold 80%.

Show FASTA Sequence

>Sequence -887
CAATGGGATTGGAAATAACAGAGACCATTGATAGTCATGGTGTAAAATAAGTGAGAAAGTACGAGGACATTATTATTTAGCTTTTGGATTTGGAATGTTAAAGAATAAGAATACCCCAATATTATTAGACTATCAGTGGTT

Display 10 profiles   Filter:

Matrix ID	Name	Score	Relative score	Sequence ID	Start	End	Strand	Predicted sequence
MA0090.2	MA0090.2.TEAD1	12.077535	0.9709691228309396	Sequence	90	99	-	AACATTCCAA
MA0090.3	MA0090.3.TEAD1	13.126196	0.920166627596659	Sequence	88	100	-	TAACATTCCAAAT
MA0090.2	MA0090.2.TEAD1	5.336632	0.8320785386069596	Sequence	108	117	+	AGAATACCCC
MA0090.1	MA0090.1.TEAD1	9.278916	0.8268933311396665	Sequence	88	99	-	AACATTCCAAAT
MA0090.2	MA0090.2.TEAD1	4.4141912	0.8130724342535866	Sequence	66	75	+	GACATTATTA
MA0090.2	MA0090.2.TEAD1	4.2522306	0.8097353734862716	Sequence	3	12	-	CCAATCCCAT

Showing 1 to 6 of 6 entries   Previous 1 Next

Comment: This type of analysis has a high sensitivity but abysmal selectivity. In other words: while true functional will be detected in most cases, most predictions will correspond to sites bound in vitro but with no function in vivo. A number of additional contraints of the analysis can improve the prediction; phylogenetic footprinting is the most common. We recommend using the ConSite service, which uses the JASPAR datasets. The review Nat Rev Genet. 2004 Apr;5(4):276-87 gives a comprehensive overview of transcription binding site prediction
